# Comparing the quality of care for long-term ventilated individuals at home versus in shared living communities: a convergent parallel mixed-methods study

**DOI:** 10.1186/s12912-022-00986-z

**Published:** 2022-08-11

**Authors:** Hanna Klingshirn, Laura Gerken, Katharina Hofmann, Peter Ulrich Heuschmann, Kirsten Haas, Martha Schutzmeier, Lilly Brandstetter, Thomas Wurmb, Maximilian Kippnich, Bernd Reuschenbach

**Affiliations:** 1 Catholic University of Applied Sciences Munich, Preysingstraße 95, D-81667 München, Germany; 2 Institute for Clinical Epidemiology and Biometry, Julius-Maximilian University Würzburg, Josef-Schneider-Straße 2, D-97080 Würzburg, Germany; 3 Clinical Trial Center Würzburg, University Hospital Würzburg, Josef-Schneider-Straße 2, D-97080 Würzburg, Germany; 4 Comprehensive Heart Failure Center Würzburg, University and University Hospital Würzburg, Am Schwarzenberg 15, D-97078 Würzburg, Germany; 5 Department of Anaesthesiology, Intensive Care, Emergency and Pain Medicine, University Hospital Würzburg, Oberdürrbacher Straße 6, D-97080 Würzburg, Germany

**Keywords:** Home mechanical ventilation, Quality of care, Living situation, Person-centred care, Mixed-methods

## Abstract

**Background:**

People on home mechanical ventilation (HMV) belong to a heterogeneous population with complex care needs. In Germany, outpatient intensive care is provided in people's private home (PH) or in shared living communities (SLC). Increasing patient numbers have led to criticism of the quality of care in recent years. Since quality deficits from the perspective of those affected are largely unclear, the following research question emerged: How do interviews with ventilated individuals and family caregivers explain any differences or similarities in the quality of care between PH and SLC?

**Methods:**

This study used a mixed-methods convergent parallel design, where quantitative and qualitative components were separately collected and analysed. The quantitative component (structured interviews and online survey) included ventilation characteristics, health-related resource use, health-related quality of life (HRQL) measured with the Severe Respiratory Insufficiency Questionnaire (SRI; range 0-100; higher scores indicated higher HRQL) and the Burden Scale of the Family Caregivers short version (BSFC-s; range 0-30; higher scores indicated higher burden). The qualitative component (semi-structured interviews) focused on people's experience of person-centred care. Data were merged using a weaving method and the Picker framework of Person-Centred Care.

**Results:**

The quantitative component revealed that ventilated individuals living in PHs were on average 20 years younger than participants living in SLCs (*n* = 46; PH: 46.86 ±15.40 years vs. SLC: 65.07 ±11.78 years; *p* = .001). HRQL (*n* = 27; PH: 56.62 ±16.40 vs. SLC: 55.35 ±12.72; *p* > .999) and the burden of family caregivers (*n* = 16; PH: 13.20 ±10.18 vs. SLC: 12.64 ±8.55; *p* > .999) were not significantly different between living situation. The qualitative component revealed that person-centred care is possible in both care settings (ventilated individuals: *n* = 13; family caregivers: *n* = 18).

**Conclusion:**

This study describes a care situation that is as heterogeneous as the population of people with HMV. HRQL and the burden of family caregivers are highly individual and, like person-centred care, independent of the living situation. Policy decisions that facilitate person-centred care need to recognise that quality of care is highly individual and starts with the free choice of the care setting.

**Supplementary Information:**

The online version contains supplementary material available at 10.1186/s12912-022-00986-z.

## Background

Home mechanical ventilation (HMV) is established treatment strategy for people with chronic respiratory failure [1]. In 2005, the prevalence of HMV per 100,000 population was 6.6 in Europe [2]. Recent studies indicated that demographic changes and advances in medical technology have increased the use of HMV worldwide [3–6]. In Germany, the number of patients hospitalised for the initiation or follow-up of HMV doubled between 2008 and 2018, with the highest increase in the 75+ age group [6]. Since HMV is extremely resource- and cost-intensive, this increasing prevalence poses enormous challenges to the health care system and influences the delivery of care [3, 7, 8].

People on HMV comprise a very heterogeneous population. Chronic respiratory failure can develop in people with chronic obstructive pulmonary disease (COPD), neuromuscular disorder (NMD), obesity hypoventilation syndrome, or thoracic-restrictive lung disease [9]. Mechanical ventilation treatment can be invasive via tracheostomy or non-invasive (NIV: non-invasive ventilation) via a mask [10]. Since many people on NIV manage their ventilation themselves, the care needs of these people are frequently lower than those of people on invasive ventilation [11]. An expanding group within the HMV population can be characterised by multiple internal and neurological comorbidities, advanced age (i.e., > 60 years), and complex care needs [3]. These people cannot be weaned from mechanical ventilation after long and severe treatment in the intensive care unit (ICU). The main criticism here is that these patients are often discharged directly from the ICU to HMV without any follow-up rehabilitation in a specialised weaning centre [12]. In addition to the fact that weaning potentials have to be clarified, it is also important to examine the indication for HMV from an ethical perspective. This means that patients and their relatives must be clearly informed about life expectancy and quality of life in the context of individual patient wishes and autonomy [10]. Another crucial group represents people with NMDs (e.g., amyotrophic lateral sclerosis, Duchenne muscular dystrophy, or spinal cord paralysis), who have little or no weaning potential due to their disease [9]. Nevertheless, this population is able to live with their families and lead a self-determined life with high participation and quality of life [13]. People with NMDs comprise a comparatively young population, which has increased only marginally over the years [3].

The practices and policies regarding HMV widely differ across countries [14]. In Germany, people with intensive care needs and HMV use can be cared for in outpatient or inpatient settings. The choice of the care setting depends on the affected individual (or the person's legal guardian) and is also influenced by the severity of the underlying disease, the complexity of individual care needs, structural needs such as accessibility, and the regional availability of outpatient intensive care services. Inpatient care is provided in specialised intensive care facilities. Outpatient intensive care can occur in a person’s private home (PH) or in a shared living community (SLC). At PHs, affected individuals are usually provided with 24/7 care by specialised intensive care services (i.e., skilled nurses) or by assisted care (i.e., lay helpers, assistants or family caregivers). This care model enables a very high degree of autonomy and participation [10]. SLCs are self-determined communities with a maximum of 12 residents, whereby the care service providers can be freely chosen and have guest status. Compared to individual intensive care, SLCs are less staff-intensive and therefore more lucrative for providers and place less burden on the health care system [15].

Currently, in Germany, there is much criticism about the quality of care for people on HMV regarding financial disincentives, unmet quality standards, and a lack of control mechanisms [16, 17]. In response, the German government passed the Intensive Care and Rehabilitation Strengthening Act (IPReG) in 2020 [18]. Although the IPReG promotes necessary regulations, such as the assessment of weaning potential, simplified access to rehabilitation and mandatory quality controls, it is under massive criticism for disregarding the heterogeneous needs of the affected individuals and for violating their rights of self-determination. According to the IPReG, expensive outpatient intensive care should only be approved under strict conditions, while the more cost-saving inpatient care should become standard care [18].

Well-defined quality standards must be created to reform care structures and ensure that every person on HMV receives care that meets their individual needs. Quality of care is a complex and multifaceted construct, and studies evaluating quality of care should aim to capture it in a multidimensional way [19]. The US Institute of Medicine suggests that improvements in quality of care should focus on six dimensions: care should be safe, effective, person-centred, timely, efficient and equitable [20]. Therefore, person-centred care is recognised as a key component of developing high-quality care [21]. It has also been recommended to compare the perceived quality with the clinical quality of care [19]. Perceived quality of care can be understood as the patient's “subjective” and “dynamic” perception of “the extent to which expected health care is received” [22]. Clinical quality of care is defined by “the interaction between health care providers and patients” and describes how inputs from a health care system could be transformed into health outcomes [19].

There are some studies focusing on specific aspects of the quality of care for individuals on HMV. Some qualitative studies deal with safety identified care deficits such as competence gaps of health care professionals or difficulties in building relationships with patients and family caregivers [23–25]. Quantitative studies found that especially in invasive HMV patients who have failed prolonged weaning health-related quality of life (HRQL) was severely impaired [13, 26]. Moreover, older patients with COPD and multiple comorbidities were likely to have a lower HRQL than patients with neuromuscular diseases [26]. COPD patients with long-term NIV had a lower HRQL if they were severely care-dependent and lived in a nursing home [27]. A single qualitative study found that person-centred care practise during prolonged weaning from mechanical ventilation could optimise weaning success [28]. Further studies indicate the high burden on the families of HMV patients [29–31], whereby the large gap between the expectations of the families and the actual support services became apparent [29]. Overall, there is limited evidence on how HMV should be delivered to affected individuals to improve person-centred outcomes [14]. Therefore, the project ‘Optimising the Care of Ventilated Patients in the Outpatient Intensive Care’ (German acronym: OVER-BEAS) was recently initiated in Bavaria, Germany [32]. One objective of the project was to examine the quality of care for people on HMV from the perspective of health care professionals [33]. In the current part of the project, the perspective of the affected individuals and their family caregivers was examined. In this study, quality of care was examined as a multifaceted construct (i.e., clinical and perceived quality of care) that considered the different living conditions in outpatient intensive care with a mixed-methods approach.

### Research questions

The study was guided by the following research questions:

1. Quantitative component: Are there differences in the clinical quality of care between PH and SLC?
2. Qualitative component: Are there differences in the perceived quality of care between PH and SLC?
3. Mixed-methods comparison: How do interviews with ventilated individuals and family caregivers explain any differences or similarities in the quality of care between PH and SLC?

## Methods

### Overall study design

This study used a mixed-methods convergent parallel design, where both quantitative and qualitative components were separately collected and analysed, with the purpose of merging and comparing the results [34]. Mixed-methods approaches can be particularly valuable in quality of care research since they have the potential to examine the multiple dimensions of quality in an objective (i.e., quantitative) and a subjective (i.e., qualitative) manner [35]. The convergent parallel design enables the research question to be examined from multiple perspectives. While quantitative results tend to show general trends and relationships, qualitative results provide an in-depth personal perspective of the affected individuals. The combination of these two types of results provides a more complete picture of the care situation than either dataset alone [36].

The widely recognised Picker Principles of Person-Centred Care were used as an overarching framework for our mixed-methods study, as they address every facet of care across the patients’ pathway [21]. Defined by eight principles the framework provides essential key elements for the delivery of high-quality care (Picker Institute Europe: Picker Principles of Person Centred Care, unpublished):

1. Fast access to reliable health advice
2. Effective treatment delivered by trusted professional
3. Continuity of care and smooth transition
4. Involvement in decisions and respect for preference
5. Clear information, communication, and support for self-car
6. Involvement of, and support for, family and carer
7. Emotional support, empathy and respect
8. Attention to physical and environmental needs

**Fig. 1:**
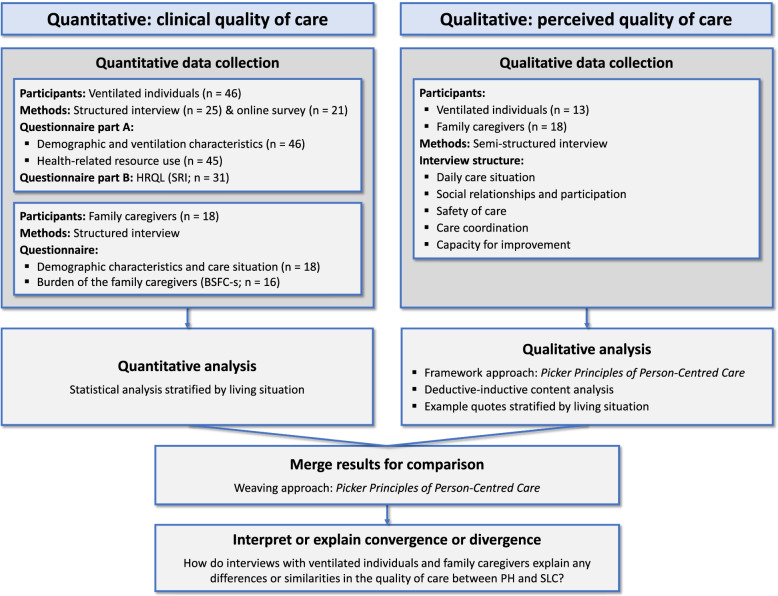
Process of the convergent parallel mixed-methods study. *Abbreviations:* *BSFC-s* Burden Scale for Family Caregivers – short, *PH* private home, *SLC* shared living community, *SRI* Severe Respiratory Insufficiency questionnaire

For the quantitative component, the clinical quality of care was described using data on ventilation, health-related resource use, HRQL and burden of the family caregivers. For the qualitative component, the perceived quality of care was examined using the Picker Principles of Person-Centred Care [21, (Picker Institute Europe: Picker Principles of Person Centred Care, unpublished)]. For structure and transparency, this paper used the guidance of Good Reporting of a Mixed Methods Study (GRAMMS) [37]. The process for this convergent parallel mixed-methods study is shown in Fig. 1.

### Participants and setting

Participants (i.e., ventilated individuals and their family caregivers) were recruited via intensive care services and via snowball sampling using social media and patient associations in Bavaria, Germany.

Intensive care services listed in an official register were invited to support recruitment of study participants via mail and a subsequent telephone call. Intensive care services willing to support our study acted as gatekeepers for recruitment. Participants (i.e., ventilated individuals) were eligible if they met the following inclusion criteria: adult (i.e., at least 18 years old), diagnosis of chronic respiratory failure, invasive or NIV, full or partial ventilator support, and living in an outpatient care setting (i.e., PH or SLC) in Bavaria. The exclusion criterion was being in the terminal stage of disease (i.e., end-stage disease). The inclusion criteria for family caregivers were being an adult (i.e., at least 18 years old) and being involved in the care or support of the ventilated individual. For data protection reasons, the intensive care services provided written study information to potential study participants (or their legal guardians). Potential study participants were encouraged to contact the study team if they had any further questions. Contact details were not shared until the potential participant gave informed written consent for study participation.

Potential participants who were reached personally via snowball sampling (i.e., study information via social media or patient associations) were invited to contact us directly. When contact was made, eligibility screening was conducted by the research team.

Participants of the online survey were also recruited via social media and patient associations, whereby the inclusion criteria were reviewed prior to the actual survey.

### Overall data collection procedure

Data collection was carried out by semi-structured face-to-face (or telephone) interviews (qualitative), structured face-to-face (or paper-pencil) interviews (quantitative), and an online survey (quantitative). Except for the online survey, qualitative and quantitative data collection was planned during one visit in one care situation, adapting the approach according to the needs of the families. Each participant was assigned an identity number (ID) to allow data pseudonymisation. Questions were answered by ventilated individuals and their family caregivers with the support of nursing staff or medical records. All interviewers were trained in a structured, half-day workshop conducted by members of the research team (HK and LG) and had a professional background (i.e., skilled nurses, occupational therapist and speech therapist) which was necessary to respond sensitively to the participants.

To adapt the interview situation to the capabilities of the ventilated individuals, the cognitive and communicative status was clarified prior to data collection with the responsible contact persons. In order to include the ventilated individuals actively and within their capabilities in the research, the quantitative part of the data collection was divided into part A (i.e., demographics, ventilation characteristics and health-related resource use) and part B (i.e., HRQL). Ventilated individuals with severely limited cognitive and communicative abilities were eligible to participate in part (A) of the quantitative study represented by a proxy (i.e., family caregiver or primary nurse). Ventilated individuals without cognitive impairment but with limited communicative abilities could also answer the HRQL measure (parts A and B). Participation in the quantitative and qualitative study was possible if the ventilated individual had the cognitive and communicative abilities to conduct a full interview (i.e., German or English language skills, communication skills beyond yes and no answers, no severe aphasia or dysarthria, and no cognitive impairment). The use of special communication aids and strategies was welcomed (e.g., written interviews and eye tracking computer devices). Family caregivers could describe their perspective of the care situation in a qualitative interview, either in addition to the ventilated individual or independently of him or her. Furthermore, quantitative data was collected from the caregivers to describe demographics, care situation and burden of the family caregivers. To keep the effort of the participants as low as possible, family caregivers were offered to conduct their interviews by telephone as an alternative to face-to-face interviews. The interview was scheduled as soon as a person agreed to participate in the study.

### Quantitative data collection

Quantitative data collection started in June 2019. Data were collected with structured face-to-face interviews based on different measures. Since in the first COVID-19 wave, extensive contact bans were adopted in Germany in mid-March 2020 [38], face-to-face data collection was no longer possible. Quantitative data collection was therefore continued as an online survey. Participants were invited to take part in the survey via social media and patient associations. This change was approved by the responsible ethics committee with an amendment. The online survey started in May 2020 and was concluded in August 2020 (i.e., end of quantitative data collection).

#### Measures for the ventilated individuals

##### Demographic and ventilation characteristics

Demographics (i.e., gender, age, and living situation) and medical history of mechanical ventilation (i.e., underlying disease, type of ventilation, duration of ventilation in years, and spontaneous breathing) were collected to describe the participants’ characteristics.

##### Health-related resource use

Health-related resource use was measured with questions regarding nursing care, therapeutic and medical care, ventilation equipment, and medical aids and technical devices. The questionnaire of the Safety in Home Care for Ventilated Patients (SHAPE) study was used as a template for the development of the questions [23]. Long-term care needs were defined by the specific long-term care assessment of individual care needs of the Medical Service of the Health Insurance Funds (MDK), resulting in five care grades [39].

##### Health-related quality of life

HRQL was measured with the Severe Respiratory Insufficiency questionnaire (SRI) [40]. The SRI is a valid, condition-specific questionnaire for people with severe respiratory insufficiency receiving HMV. Currently, the SRI is the most widely used international instrument for assessing HRQL in people with various chronic respiratory disorders [41]. The SRI contains 49 items, each of which was rated on a 5-point Likert scale (‘strongly disagree’ to ‘strongly agree’). The SRI comprised seven independent subscales (respiratory complaints, physical functioning, attendant symptoms and sleep, social relationships, anxiety, psychological well-being, and social functioning). Each subscale produced a score between 0 and 100, with higher scores indicating higher HRQL. The subscales were aggregated to one summary score.

#### Measures for the family caregivers

##### Demographic characteristics and care situation

Information about demographics (i.e., gender, age, and relationship to the ventilated individual) and the care situation (i.e., shared household living or involvement in specialised nursing) was collected prior to the qualitative interviews.

##### Burden of the family caregivers

Family caregivers who participated in the qualitative interview additionally provided information about their subjectively experienced burden. This was measured with the short version of the Burden Scale for Family Caregivers (BSFC-s) [42]. The BSFC-s is a valid 10-item assessment, with each item rated on a 4-point Likert scale (‘strongly disagree’ to ‘strongly agree’). A higher score indicates a higher subjective burden for the relative: low burden (0-4), moderate burden (5-14), and high burden (15-30) [43].

#### Qualitative data collection

Qualitative data were collected from June 2019 to March 2020 using semi-structured interviews. Prior to the interview, the participants were informed about the background, aim and procedure of the study. To avoid overloading, it was emphasised that breaks are possible at any time during the interview. The participants were encouraged to be critical and reflective and present their individual perception of the care situation. In addition to informed written consent, all participants provided verbal informed consent before the audio recording of the interview. The interview guide started with an opening question, which transitioned to the following five key questions: (1) daily care situation, (2) social relationships and participation, (3) safety of care, (4) care coordination, and (5) capacity for improvement. These five areas were chosen to present a broad view of the perceived quality of care and to address the entire care pathway in the spirit of the Picker Principles. Key questions were the same for ventilated individuals and family caregivers, but follow-up questions or probes (i.e., further explanations or examples offered when additional information was necessary) were adapted to the respective participant group. To close the interview, the participants were asked if there was anything else to tell. Field notes (e.g., interview duration and special incidents) were taken after the interview. The structure of the interview is presented in Table 1.

**Table 1:** Interview structure

Focus	Key questions	Follow-up questions for ventilated individuals	Follow-up questions for relatives
Daily care situation	Please describe your typical daily routine. Start in the morning. What happens first when you wake up?	Where in everyday life do you need support? Who assists you? Imagine washing and dressing, eating and drinking, moving around or toileting. What are the problems in your daily care? What works well?	What are your responsibilities as a family caregiver? What are the problems in daily care? What works well? In your role as a family caregiver: What do you find fulfilling? What is less fulfilling, exhausting or difficult? Please, give an example.
Social relationships and participation	What is your experience of everyday life with friends or family?	What role do care services or relatives play in this? What do you need to be independent? Imagine meeting friends, trips, or holidays. How do you experience the quality of your social relationships? Please, illustrate this.	What changes in social life have you experienced? How do you experience the quality of your social relationships? Please, illustrate this.
Safety of care	What role does safety of care play for you?	Please share a situation that made you feel unsafe. What exactly happened?	Please share a situation that made you feel unsafe. What exactly happened?
Care coordination	How is care organised?	Who coordinates the contacts? How and with whom is communication organised? How is the collaboration between the involved professionals organised? What is your role in this? What roles do others have? What are problems in care coordination? What works well? Please, illustrate this.	Who coordinates the contacts? How and with whom is communication organised? How is the collaboration between the involved professionals organised? What is your role in this? What roles do others have? What are problems in care coordination? What works well? Please, illustrate this.
Capacity for improvement	Where do you see a substantial need for improvement of care?	Please, summarise.	Please, summarise.

#### Pilot testing

The questionnaires for the quantitative data collection and the interview guides for the qualitative data collection were pilot tested in both care settings (PH and SLC) with two ventilated individuals. After the interview, the participants were asked for feedback on the feasibility, appropriateness and comprehensibility of the questions, the duration of the interview, and the interview situation. The pilot test provided support for the developed interview structure and did not result in major changes. The online survey also went through a pre-test phase, where it was possible to complete the questionnaire online and, if necessary, add comments to specific questions.

#### Ethics

The study including an amendment was approved by the ethics committee of the Catholic University of Applied Sciences Munich. Written informed consent was obtained from all participants before conducting the study.

#### Data analysis

##### Quantitative analysis

Discrete data are presented as absolute (n) and relative (%) frequencies. Continuous data are presented as the mean (M) ± standard deviation (SD) and were tested for normal distribution using the Shapiro-Wilk test. Group comparisons of the results were performed with respect to living in a PH versus living in an SLC (hypothesis = there is no difference between the settings PH and SLC). To estimate group effects, paired t-tests were used for normally distributed data. For nonnormally distributed data, a nonparametric test (Wilcoxon–Mann–Whitney rank sum test) was used. Group effects were tested with a 2-sided level of 0.05. For nominal data with two or more categories, Fisher’s exact test was used to compare the proportions between the PH and SLC care settings. For each sub-hypothesis (see tables), p values were corrected for multiple group comparisons based on Bonferroni–Holm tests. Further subgroup analyses were performed to compare individuals with invasive ventilation versus NIV and to compare individuals taking part in the offline (i.e., face-to-face or paper-pencil survey) versus online survey. The data analysis was performed with IBM© SPSS© Statistics, Version 24.

##### Qualitative analysis

The qualitative analysis was guided by the framework method [44], a systematic approach providing clear steps to produce highly structured outputs. As a theoretical basis for building a deductive analysis matrix, we used the Picker Principles of Person-Centred Care [21, (Picker Institute Europe: Picker Principles of Person Centred Care, unpublished)]. We used a deductive-inductive approach [45], with the eight Picker principles as deductive meta-codes and inductively built sub-codes (i.e. the sub-codes were derived directly from the interviews with the ventilated individuals and the family caregivers). The codebook for the framework analysis showing the definition for each Picker principle as well as themes and example quotes for each sub-code is provided in Additional file 1.

The qualitative interviews were audio-recorded and transcribed verbatim. For data protection personal data were pseudonymised. All participants were offered to review their transcript for quality control. Using qualitative content analysis, the transcripts were segregated into distinct manageable units or ‘meaning units’ [45]. Data were independently analysed by two researchers (HK: MPH, occupational therapist and LG: MSc, skilled nurse). Both researchers were experienced in qualitative data analysis. All interview transcripts and all independent built codes were subsequently cross-compared, whereby disagreement was discussed until consensus was reached between the two researchers. The sample size was limited based on the principle of meaning saturation (i.e., additional participants were included in the study as long as new themes emerged) [46]. Saturation was reached after 31 interviews. Data analysis was conducted with MAXQDA software, version 20.

##### Mixed-methods analysis

Subsequent to the independent analysis of the quantitative and qualitative data, the results were merged for comparison to identify areas of convergence and divergence using a weaving approach [47] in the Discussion section. Researchers with different experience levels and professional backgrounds were involved in the integration of the data (HK, LG, KaH and BR). The Picker Principles of Person-Centred Care [21, (Picker Institute Europe: Picker Principles of Person Centred Care, unpublished)] were used to structure the merging and comparing of results.

## Results

### Recruitment

Recruitment ran in parallel with data collection. Out of 136 approached intensive care services that were invited, 35 agreed to support recruitment. Within these intensive care services, 180 individuals met the inclusion criteria (PH: *n* = 89; SLC: *n* = 91). Of these, 22 (response: 12.2 %) ventilated individuals consented to participate. Another three participants attended the study by snowball sampling via patient associations, resulting in a total of 25 ventilated individuals taking part in the face-to-face (or paper-pencil) interviews. An additional 21 individuals took part in the online survey, resulting in a total of 46 ventilated individuals being included in the quantitative analysis.

### Quantitative results

#### Demographic and ventilation characteristics

The demographic and ventilation characteristics of the individuals on HMV are shown in Table 2. Of the 46 ventilated individuals, 28 (60.9 %) were male, and 28 (60.9 %) lived in their PH. The mean age was 53.99 ±16.60 years. The participants living in a PH were on average almost 20 years younger than the participants living in an SLC (PH: 46.86 ±15.40 years vs. SLC: 65.07 ±11.78 years; *p* = .001). The participants living in a PH were on ventilation an average of more than eight years longer than the participants in an SLC (*p* < .001). Of the ventilated individuals, 13 participated in the qualitative interviews (see Table 2, column 2). Since one of the interview participants lived in two different care settings (PH and inpatient setting), we excluded the person from further quantitative analysis. During the qualitative interview, the participant only referred to the care at home.

**Table 2:** Demographic and ventilation characteristics in the PH versus SLC groups

	Participants with Interview		Participants who took part in the study							
			PH		SLC		Total		***P*** value	P adjusted ^**a**^
Subjects, N	13		28		18		46			
Gender, n (%)										
Female	5	(38.5)	9	(32.1)	8	(44.4)	17	(37.0)	.718	>.999
Male	8	(61.5)	18	(64.3)	10	(55.6)	28	(60.9)		
Divers	0	0	1	(3.6)	0	0	1	(2.2)		
Age, years, M (±SD)	52.17	(±16.75)	46.86	(±15.40)	65.07	(±11.78)	53.99	(±16.60)	<.001*	<.001*
Underlying disease, n (%)									<.001*	.002*
NMD	7	(53.8)	17	(60.7)	5	(27.8)	22	(47.8)		
Spinal cord paralysis	0	0	4	(14.3)	0	0	4	(8.7)		
Central nervous system disease ^b^	2	(15.4)	0	0	8	(44.4)	8	(17.4)		
COPD	2	(15.4)	4	(14.3)	2	(11.1)	6	(13.0)		
Pneumonia	1	(7.7)	1	(3.6)	1	(5.6)	2	(4.3)		
Chest deformity	1	(7.7)	1	(3.6)	1	(5.6)	2	(4.3)		
Post-operative complications	0	0	1	(3.6)	1	(5.6)	2	(4.3)		
Type of ventilation, n (%)									.069	.207
Invasive	10	(76.9)	20	(71.4)	17	(94.4)	37	(80.4)		
NIV	3	(23.1)	8	(28.6)	1	(5.6)	9	(19.6)		
Duration of ventilation, years, M (±SD)	9.26	(±5.86)	11.67	(±8.00)	3.08	(±2.44)	8.67	(±7.76)	<.001*	<.001*
Spontaneous breathing, n (%)									.768	>.999
Ventilation < 16 h	6	(46.2)	10	(35.7)	7	(38.9)	17	(37.0)		
Ventilation > 16 h	1	(7.7)	3	(10.7)	3	(16.7)	6	(13.0)		
Continuous ventilation	6	(46.2)	15	(53.6)	8	(44.4)	23	(50.0)		

*N = 46*. Data are presented as absolute numbers (n) and relative frequencies (%) or as the means (M) and standard deviations (SD)

*Missing values:* Duration of ventilation, years (*n* = 3)

*Abbreviations: COPD* Chronic obstructive pulmonary disease, *NMD* Neuromuscular disorder, *NIV* Non-invasive ventilation, *PH* Private home,* SLC* shared living community

*Significant at *p* < 0.05

^a^Adjusted with Bonferroni–Holm correction for multiple testing

^b^Central nervous system disease, e.g., stroke, traumatic brain injury, hypoxic brain injury, subarachnoid haemorrhage

#### Health-related resource use

Data on health-related research use are presented in Table 3. Data on long-term care needs show that more than half of the participants had most severe impairments (*n* = 23; 51.1 %). Most of the participants were provided with specialised nursing care (*n* = 33; 73.3 %), whereby, particularly for those in PHs, assisted care or a combination of both types of care were also applied (*p* = .004). Almost all participants received physical therapy (*n* = 43; 95.6 %). Occupational therapy (PH: 44.4 % vs. SLC 82.4 %; *p* = .130) and speech therapy (PH: 25.9 % vs. SLC: 83.3 %; *p* = .001) were used considerably more often in SLCs. There were no differences between PH and SLC groups in the use of medical care and ventilation equipment. The use of medical aids and technical devices showed few differences. An exception was the use of a powered wheelchair (PH: 59.3 % vs. SLC: 5.9 %; *p* = .005).

**Table 3:** Health-related resource use in the PH versus SLC groups

	PH		SLC		Total		***P*** value	P adjusted ^**a**^
	n	(%)	n	(%)	n	(%)		
**Nursing care**								
Long-term care needs							.785	.785
None to considerable impairments	2	(7.4)	0	0	2	(4.4)		
Serious impairments	3	(11.1)	3	(16.7)	6	(13.3)		
Severe impairments	8	(29.6)	6	(33.3)	14	(31.1		
Most severe impairments	14	(51.9)	9	(50.0)	23	(51.1)		
Type of care							.002*	.004*
Specialised nursing care	15	(55.6)	18	(100)	33	(73.3)		
Assisted care	7	(25.9)	0	0	7	(15.6)		
Both combined	5	(18.5)	0	0	5	(11.1)		
**Therapeutic and medical care**								
Physical therapy, last 3 months	25	(92.6)	18	(100)	43	(95.6)	.509	>.999
Occupational therapy, last 3 months	12	(44.4)	14	(82.4)	26	(59.1)	.026	.130
Speech therapy, last 3 months	7	(25.9)	15	(83.3)	22	(48.9)	<.001*	.001*
Respiratory therapy, last 3 months	5	(18.5)	1	(6.7)	6	(14.3)	.395	>.999
General practitioner, last 3 months	24	(88.9)	16	(88.9)	40	(88.9)	>.999	>.999
Medical specialist, last 3 months	11	(40.7)	9	(50.0)	20	(44.4)	.559	>.999
**Ventilation equipment**								
2^nd^ ventilator	22	(81.5)	13	(76.5)	35	(79.5)	.716	>.999
Bag-valve-mask	21	(77.8)	14	(77.8)	35	(77.8)	>.999	>.999
Oxygen device	16	(61.5)	15	(83.3)	31	(70.5)	.182	>.999
Respiratory gas humidifier	12	(44.4)	13	(72.2)	25	(55.6)	.078	.544
Suction device	21	(77.8)	15	(88.2)	36	(81.8)	.455	>.999
Inhalation device	23	(85.2)	15	(83.3)	38	(84.4)	>.999	>.999
Cough assist	15	(55.6)	6	(33.3)	21	(46.7)	.223	>.999
**Medical aids and technical devices**								
Wheelchair	16	(59.3)	17	(94.4)	33	(73.3)	.014*	.157
Powered wheelchair	16	(59.3)	1	(5.9)	17	(38.6)	<.001*	.005*
Transfer aids (e.g., hoist)	19	(70.4)	11	(68.8)	30	(69.8)	>.999	>.999
Standing aids (e.g., standing frame)	2	(7.4)	4	(23.5)	6	(13.6)	.186	>.999
Bathing aids (e.g., bath seat)	12	(44.4)	8	(44.4)	20	(44.4)	>.999	>.999
Nursing care bed	22	(81.5)	18	(100)	40	(88.9)	.073	.658
Toilet aids (e.g., commode chair)	13	(48.1)	9	(52.9)	22	(50.0)	>.999	>.999
Walking aids (e.g., rollator)	4	(14.8)	6	(35.3)	10	(22.7)	.150	>.999
Positioning aids (e.g., wedges)	17	(63.0)	15	(88.2)	32	(72.7)	.090	.717
Feeding tube and pump	15	(55.6)	17	(94.4)	32	(71.1)	.006*	.078
Communication devices	8	(29.6)	6	(35.3)	14	(31.8)	.748	>.999
Adaptive computer equipment	12	(44.4)	4	(25.0)	16	(37.2)	.328	>.999
Aids for incontinence care	7	(25.9)	8	(61.5)	15	(37.5)	.041*	.412

*Missing values****:*** occupational therapy (*n* = 1), respiratory therapy (*n* = 3), 2^nd^ ventilator (*n* = 2), oxygen device (*n* = 1), suction device (*n* = 1), powered wheelchair (*n* = 1), transfer aids (*n* = 2), standing aids (*n* = 1), toilet aids (*n* = 1), walking aids (*n* = 1), positioning aids (*n* = 1), communication devices (*n* = 1), adaptive computer equipment (*n* = 2), and aids for incontinence care (*n* = 5)

*N = 45*. Data are presented as absolute numbers (n) and relative frequencies (%)

*Abbreviations: PH* Private home, *SLC* Shared living community

*Significant with *p* < 0.05

^a^Adjusted with Bonferroni–Holm correction for multiple testing (for each sub-hypothesis: nursing care, therapeutic and medical care, ventilation equipment, medical aids and technical devices)

#### Health-related quality of life

**Fig. 2:**
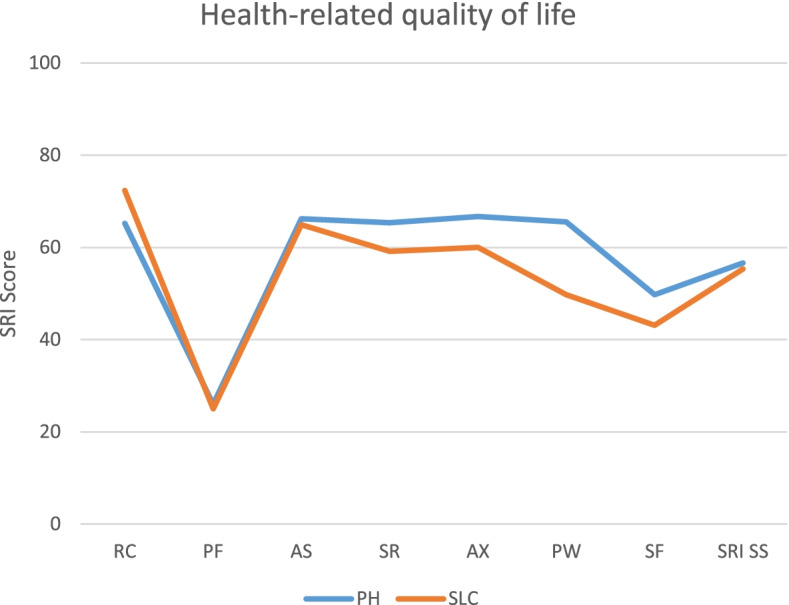
Graphical presentation of the health-related quality of life in the PH versus SLC groups. *N* = 31. Mean subscale and summary scale scores from the SRI in individuals on HMV stratified by living situation (PH vs. SLC). Higher scores indicate a higher HRQL. *Missing values:* respiratory complaints (*n* = 2), social relationships (*n* = 1), anxiety (*n* = 2), social functioning (*n* = 1), SRI summary score (*n* = 4). *Abbreviations:* *HRQL* health-related quality of life, *SRI* Severe Respiratory Insufficiency questionnaire, *HMV* home mechanical ventilation, *PH* private home, *SLC* shared living community, *RC* respiratory complaints, *PF* physical functioning, *AS* attendant symptoms and sleep, *SR* social relationships, *AX* anxiety, *PW* psychological well-being, *SF* social functioning, *SS* summary scale

The data on HRQL are presented in Table 4. The SRI was completed by 31 participants, but due to missing values, the summary score was only based on answers from 27 participants. Overall, the mean SRI summary score was 56.15 ±14.90, indicating a medium HRQL. The lowest score was on the subscale ‘physical functioning’ (25.63 ±21.21). In comparing living situations, there was no significant difference in the SRI summary score (PH: 56.62 ±16.40 vs. SLC: 55.35 ±12.72; *p* > .999). The largest difference was found in the subscale ‘psychological wellbeing’ (PH: 65.55 ±21.99 vs. SLC: 49.78 ±21.13; *p* = .500). A graphical comparison of the mean SRI scores for those in PHs versus SLCs is displayed in Fig. 2.

**Table 4:** Health-related quality of life in the PH versus SLC groups

	PH		SLC		Total		***P*** value	P adjusted ^**a**^
	M	(±SD)	M	(±SD)	M	(±SD)		
Respiratory complaints	65.23	(±22.20)	72.35	(±13.40)	67.93	(±19.38)	.291	>.999
Physical functioning	25.98	(±21.99)	25.00	(±20.75)	25.63	(±21.21)	.905	>.999
Attendant symptoms and sleep	66.25	(±20.90)	64.93	(±16.81)	65.78	(±19.27)	.859	>.999
Social relationships	65.35	(±17.46)	59.17	(±27.46)	63.08	(±21.41)	.456	>.999
Anxiety	66.71	(±24.97)	60.00	(±25.39)	64.40	(±24.87)	.500	>.999
Psychological well-being	65.55	(±21.99)	49.78	(±21.13)	59.96	(±22.67)	.063	.500
Social functioning	49.79	(±23.94)	43.10	(±23.40)	47.34	(±23.56)	.463	>.999
SRI summary score	56.62	(±16.40)	55.35	(±12.72)	56.15	(±14.90)	.835	>.999

*N = 31*. All data are presented as the means (M) and standard deviations (SD)

*Missing values:* respiratory complaints (*n* = 2), social relationships (*n* = 1), anxiety (*n* = 2), social functioning (*n* = 1), SRI summary score (*n* = 4)

*Abbreviations:* *PH* Private home, *SLC* Shared living community, *SRI* Severe Respiratory Insufficiency questionnaire

*Significant at *p* < 0.05

^a^Adjusted with Bonferroni–Holm correction for multiple testing

Additional results comparing the participants with invasive ventilation versus NIV and the participants taking part in the offline versus online survey are presented in Additional file 2.

#### Demographic characteristics, care situation and burden of the family caregivers

Data on the characteristics and burden of family caregivers are presented in Table 5. A total of 18 family caregivers participated in the study. Ten family caregivers were female (55.6%), and the mean age was 52.06 ±12.34 years. Overall, the mean BSFC-s summary score was 12.81 ±8.74, indicating a moderate burden on the family caregivers. There was no significant difference in the BSFC-s summary score between family caregivers caring for someone in a PH and those caring for someone in an SLC (PH: 13.20 ±10.18 vs. SLC: 12.64 ±8.55; *p* > .999).

**Table 5:** Characteristics and burden of the family caregivers in the PH versus SLC groups

	PH		SLC		Total		***P*** value	P adjusted ^**a**^
Subjects, N	5		13		18			
Female, n (%)	4	(80.0)	6	(46.2)	10	(55.6)	.314	>.999
Age, years; M (±SD)	52.80	(±5.36)	51.75	(±14.52)	52.06	(±12.34)	.879	>.999
Relationship to the VI, n (%)							.022	.135
Spouse or partner	2	(40.0)	5	(38.5)	7	(38.9)		
Mother or father	3	(60.0)	0	0	3	(16.7)		
Son or daughter	0	0	5	(38.5)	5	(27.8)		
Other	0	0	3	(23.1)	3	(16.7)		
Shared household living, n (%)	5	(100)	0	0	5	(27.8)	<.001*	<.001*
Relatives involved in nursing, n (%)	2	(40.0)	0	0	2	(11.1)	.065	.327
Burden of relatives, n (%)							>.999	>.999
Low burden BSFC-s (0-4)	1	(20.0)	3	(27.3)	4	(25.0)		
Moderate burden BSFC-s (5-14)	2	(40.0)	3	(27.3)	5	(31.3)		
High burden BSFC-s (15-30)	2	(40.0)	5	(45.5)	7	(43.8)		
BSFC-s summary score, M (±SD)	13.20	(±10.18)	12.64	(±8.55)	12.81	(±8.74)	.910	>.999

*N = 18*. Data are presented as absolute numbers (n) and relative frequencies (%) or as the means and standard deviations (SD)

*Missing values:* age, years (n = 1), burden of relatives and BSFC summary score (*n* = 2 each)

*Abbreviations:* *BSFC-s* Burden Scale for Family Caregivers – short, *PH* Private home, *SLC* Shared living community, *VI* Ventilated individual

*Significant at *p* < 0.05

^a^Adjusted with Bonferroni–Holm correction for multiple testing

### Qualitative results

Thirty-one participants took part in the qualitative interviews: thirteen ventilated individuals (see Table 2) and 18 family caregivers (see Table 5). Interviews were usually conducted as face-to-face interviews. Of the interviews with ventilated individuals, two interviews were conducted in writing due to the use of alternative and augmented communication devices. Four interviews with family caregivers were conducted as telephone interviews for pragmatic reasons. The mean duration of the interviews with ventilated individuals was 25 minutes (range: 10 to 65 minutes). The mean duration of the interviews with family caregivers was 28 minutes (range: 13 to 55 minutes). One interview with a relative had to be repeated due to recording problems. Seventeen participants reviewed their transcripts, and no one made corrections.

#### Analysis of the interviews

For each Picker principle, two to four sub-codes were inductively built. In total, 742 meaning units were coded (range: 4 to 115 per sub-code). The following section describes the themes that emerged from the interviews with ventilated individuals and their family caregivers for each Picker principle. Example quotes including the participants ID and the corresponding paragraph (§) in the interview transcript were given as link to empirical data. The code system, including the number of meaning units, is presented in Table 6. The qualitative results are described narratively and illustrated with an example quote below. Further example quotes for each sub-code and both care settings (PH and SLC) are presented in Additional file 1.

1. ***Fast access to reliable health advice***

The participants in both care settings (i.e., PH and SLC) emphasised the importance of an appropriate health care network, defined by access to the right support, from the right person, at the right time. For people on HMV, this includes known and trusted advisers, support from an interprofessional team and a 24-hour emergency service. However, the participants often experienced the opposite: interprofessional outpatient medical centres for people with HMV are extremely rare. People’s care journey frequently starts with a misguided transition followed by a desperate search for a suitable care setting accompanied by an ongoing search for qualified physicians and therapists. In addition, the participants described the continuing fight for high-quality care as extremely exhausting. Knowledge, expertise, perseverance and strength are necessary for getting what is needed. A quote about the provision of respiratory equipment clarifies this point:

*Certain areas [are] paid only as a lump sum by the health insurance. [...] The quality [...] [is] getting significantly worse [...] and [...] sometimes [you] really have to fight [...]. And not all family caregivers, not all patients can fight. (Family caregivers, PH, ID14, §91)*

**Table 6:** Person-centred care for people on home mechanical ventilation

Picker principles (deductive)	Sub-codes (inductive)	n ^a^
**1. Fast access to reliable health advice**	• Being integrated in a safe health care network	13
	• Coping with inappropriate health care structures	4
	• Fighting for a high-quality care	24
**2. Effective treatment delivered by trusted professionals**	• Feeling comfortable with the personal care situation	115
	• Feeling between hope and reality – exploiting rehabilitation potentials	10
	• Feeling insecure due to staff and skills shortage	39
**3. Continuity of care and smooth transitions**	• Moving into a world of uncertainty	4
	• Welcoming visitors in the safety of the own home	9
	• Going on a care journey in good company	13
**4. Involvement in decisions and respect for preferences**	• Balancing dependence and independence	57
	• Feeling accepted with own needs and preferences	41
	• Enabling time for relationships with family and friends	34
**5. Clear information, communication, and support for self-care**	• Being supported in developing knowledge and confidence	17
	• Being alone with questions and decisions	14
**6. Involvement of, and support for, family and carers**	• Being ripped out of life and returning to normal	19
	• Caring hand in hand	63
	• Being part of a family – being cared for	36
	• Dealing with burdens and challenges	69
**7. Emotional support, empathy and respect**	• Blurring boundaries	6
	• Living with a shadow – living with assistance	15
	• Feeling isolated and defenceless	11
**8. Attention to physical and environmental needs**	• Communicating and being understood	12
	• Dealing with complex care needs and complex planning	34
	• Improving participation through technology	37
	• Balancing safety and living an active life	46

^a^Number of meaning units per sub-code

2. ***Effective treatment delivered by trusted professionals***

Independent of the care setting, most of the participants felt comfortable with their personal care situation. This comfort was based on a trusting relationship between staff, ventilated individuals, and their families. Moreover, person-centred care should be guided by the idea of assistance and tailored to individual needs. This becomes clear in the following quote:

*So, what is important to me about the nurses is - and most of them [...] also have this - patience, time, listening, doing what I say and doing things the way I need them and not the way they are used to or someone else dictates. (Ventilated individual, PH, ID03, §13)*

The participants presumed that an effective treatment has to include long-term rehabilitation and support in developing realistic and person-centred treatment goals. Staff and skill shortages were stated as a main problem in realising effective treatment. Growing deficits in the number of skilled professionals, high staff turnover, and an increasing number of foreign-educated nurses (including associated cultural and language barriers) was reported as a serious gap in safety of care.

3. ***Continuity of care and smooth transitions***

The participants described the initiation of HMV as a step into a world of uncertainty. To make people feel safe in this new and sometimes frightening life situation, being accompanied during the transition was pointed out as crucial. To ensure an overall good experience during the care journey, this transition should be initiated in the discharging centre (optimally a specialised ventilation centre). In contrast, the quote below shows that hospital discharge is often unstructured and accelerated:

*We had to leave [the clinic] at that time, and that was [...] the first slap in the face. They said: in 14 days we need a place to care for our father. [...] But they said they had already called everywhere [...] but there was nothing available for him. [...] And we were pressed for time, because [the clinic] told us that if we couldn't find anything, they would find us something. He has to leave. (Family caregiver, SLC, ID31, §14-19)*

Furthermore, of the participants in both care settings stated the need for specialised outpatient treatment centres and medical home visits to avoid complex and hazardous transport and stressful hospital stays. In this context, continuity of care was reported as central and should be realised by the companionship of known and trusted professionals. This companionship should not be interrupted even during an inpatient hospital stay to guarantee the safety of care.

4. ***Involvement in decisions and respect for preferences***

The participants described living with HMV as an act of balancing dependence and independence. In both care settings, this included the intention of living a normal life despite the dependence on machines and people, making their own decisions, balancing benefits and risks and being accepted as a person with their own needs and preferences. The acceptance of one’s own needs and preferences and the naturalness of enabling time with family and friends were noted as basic conditions for successful person-centred care. The quote from a ventilated individual illustrates how activities and participation can be realised in outpatient intensive care:

*We meet once a week, in the afternoon, and talk about what we're going to eat the next week [...]. And then we can write our shopping list. Then we go shopping. I always come along [...]. And we are allowed to decide for ourselves what the service staff cook for us [...]. (Ventilated individual, SLC, ID06, §145)*

5. ***Clear information, communication, and support for self-care***

Independent of the care setting, support in developing knowledge and confidence by a trusted advisor was reported as a central aspect of informed decision-making. This requires unrestricted access to individualised and tailored information at every stage of the care journey. However, empowerment for informed decision-making is not a matter of course. The participants stated that they often felt alone with questions and decisions. It was unclear who they could turn to, and the existing support services (e.g., those from the health insurance companies) were perceived as insufficient. The quote from a relative shows a positive example for support during discharge from the hospital:

*When [my father] was to be discharged, the social services at the hospital helped me a lot with the formalities and told me that there were SLC specialised for intensive care, which I didn't know before. (Family caregiver, SLC, ID24, §87)*

6. ***Involvement of, and support for, family and carers***

The participants reflected that the critical illness of a close family member changes more than just the personal life situation of the sick person; it changes the entire family situation. The uncertainty caused by the diagnosis and the ventilation situation raises many questions. The family first has to reorient to and cope with the new situation. Therefore, the participants in both care settings emphasised the urgent need for professional companionship and involvement of the family. The participants pointed out that this needs clear roles and responsibilities, including the will of the relative to hand over control and care together. Usually, family members take responsibility for their relative as matter of course. Independent of the care setting, family caregivers experienced their caring responsibility as emotionally stressful. Care determines daily life and is associated with high workload, bureaucracy, lack of flexibility, change in social life, financial burden and restrictive housing conditions. Due to physical strains, emotional burdens, or fears and worries, the family caregivers often felt vulnerable and powerless. Therefore, the support of the family members and the ability to speak to the staff about worries were important. The following quote presents an example of family caregivers caring hand in hand with the nursing staff:

*[The coordination of care] is collaborative: [...] We as relatives [...] with the nurse. [...] If certain physicians [...] drop out, then we start searching again. [...] The [nursing staff] tend to make the appointments, because they are in everyday life, they know how it fits best for them. And then it's always a symbiosis. (Family caregiver, ID31, §61-63)*

7. ***Emotional support, empathy and respect***

The participants described role definition as a challenge in outpatient intensive care. Particularly, in a person's PH, boundaries are blurred, and nurses take on an ambiguous role between guest and family member. Independent of the care setting, outpatient intensive care must be delivered with empathy, respect and understanding with regard to the ventilated individuals and their families. In contrast, the participants reported situations where respectful care was missing, and they felt isolated and defenceless. Twenty-four-hour intensive care has a profound impact on the life and privacy of the affected individual and their family. A participant described living with assistance as ‘living with a shadow’:

*[My social relationships are] actually quite good, but also [limited], so I would say 85 % good [...]. But because I always have a shadow behind me, a nurse or someone else, it is of course hindered. (Ventilated individual, PH, ID01, §41)*

8. ***Attention to physical and environmental needs***

Independent of the care setting, physical care should comfort ventilated individuals. The participants stated that this starts with the ability to communicate and be understood. Since ventilated individuals are frequently limited in their communication, it is especially important to listen, be patient and pay attention to physical signals. Nonverbal and technology assisted communication is also important. The participants noted that the needs of ventilated individuals are complex, and since they live in a highly technical environment, every activity requires complex planning. An accessible environment and the use of technical aids can enable ventilated individuals to participate in social life. In the following quote, a participant illustrates that there should be a balance between safety and living an active life:

*[There are], of course, a variety of ‘risks’ - starting [...] with a sudden failure of the ventilation on the way, up to some barriers - actually small, but nevertheless insurmountable with the wheelchair - that ultimately force you to turn around. But: ‘No risk- no fun’. (Ventilated individual, PH, ID02, §49-50)*

#### Mixed-methods comparison

Overall, there was no significant difference between the SRI summary scores of ventilated individuals living at home compared to ventilated individuals living in SLCs. Rather, the broad range of the SRI summary scores shows that HRQL is perceived highly individual by the participants. Comparable results were found for the burden on family caregivers. Here, the BSFC-s summary scores also showed a wide range, indicating a highly individual perception of burden. Our qualitative results showed that – independent of the care setting – most of the participants ‘feeling comfortable with their personal care situation’ (115 meaning units), and high-quality person-centred care was possible in both care settings. Although no clear differences in the clinical quality of care were found in the domains of HRQL and burden of family caregivers, it appears that different populations live in the two care settings.

The quantitative component showed that the ventilated individuals living in a PH were on average 20 years younger than the ventilated individuals living in an SLC. Moreover, people living in PHs were often affected by NMD or spinal cord paralysis, have been ventilated at home since childhood or young adulthood (duration of ventilation in years: 11.67 ±8.00), and were able to live a self-determined life with high participation (qualitative results). In contrast, people living in SLCs were often long-term ventilated after stroke, traumatic or hypoxic brain injury or subarachnoid haemorrhage, have been ventilated for less time (duration of ventilation in years: 3.08 ±2.44) and were already located in the final stages of a normal lifespan. This can be seen as a clear indication that we are dealing with two very different subgroups of patients.

This assumption was reinforced by our qualitative component. The people living in SLCs and their family caregivers attached great importance to the safety aspect; they appreciated care in a highly specialised facility with a competent team and a high nursing staff ratio. It was noticeable that the term “the facility” was repeatedly used, which illustrates the proximity of SLCs to inpatient care. In contrast, people living in PHs frequently lived with their families, studied or worked and used the support of an assistant to lead a self-determined life. Even though person-centred care is possible in both care settings, the free choice of the care setting seems to have a strong influence on how the quality of care is perceived.

## Discussion

This study examined differences and similarities in the quality of care at home compared to that in an SLC for ventilated individuals and their family caregivers using a mixed-methods convergent parallel design. To understand the complex and multifaceted construct of quality of care, we compared the perceived quality (qualitative) with the clinical quality of care (quantitative) and found a high convergence between our results.

1. ***Fast access to reliable health advice***

Overall, our results showed that quality of care of the individuals on HMV was diverse and independent of the setting. The participants reported in the qualitative interviews that it was particularly difficult to find specialised occupational and speech therapists for home care. This was confirmed by our quantitative data (physical therapy: PH: 92.6 % vs. SLC: 100 %; occupational therapy: PH: 44.4 % vs. SLC: 82.4 %; speech therapy: PH: 25.9 % vs. SLC: 83.3 %). Other studies have also shown that physical therapy is used more often in individuals on HMV than occupational and speech therapy [7, 26]. Due to the complex care needs of ventilated individuals in both care settings, it seems rather unlikely that the differences between the PH and SLC groups were due to the indication alone. One further explanation could be that it is more lucrative for therapists to treat several patients within an SLC than to take a long journey for a single home visit. Another explanation is offered by our qualitative results: ventilated individuals who live at home are often more active or are working or studying and therefore simply did not have the time for many forms of therapy.

Regarding access to reliable health advice, the participants emphasised in the qualitative interviews that they had to fight for high-quality care, which was experienced as extremely burdensome. Here, the participants mainly referred to communication with health insurance providers when approving the financing of medical aids and assistive technologies. It was previously described in a Norwegian study in 2011 that care is experienced as a fight against the system [29]. An urgent recommendation would be to reduce bureaucracy and provide low-threshold access to necessary aids to relieve the burden on family caregivers.

2. ***Effective treatment delivered by trusted professionals***

The qualitative findings from our study showed that independent of the care setting, most of the participants felt comfortable with their personal care situation (115 meaning units). Moreover, the quantitative findings revealed that all ventilated individuals living in SLCs received specialised nursing care, while ventilated individuals living in PHs also received assisted care or a combination of both. Merging these findings, we see that both models of care, which are also described in the German national HMV guidelines [10], can be of high quality and therefore have their justification. In line with other studies [33, 48], our qualitative component revealed that staff and skill shortages were a main problem in outpatient intensive care. Staff and skill shortages jeopardize the sense of safety of the family caregivers and ventilated individuals and reduce trust in health care professionals [23, 24]. The introduction of Advanced Nursing Practice (ANP) in HMV could be recommended to counteract this problem and support the existing skill-grade mix.

3. ***Continuity of care and smooth transitions***

In agreement with other studies [33, 49], we found that hospital discharge often occurred in a hurried, unstructured and guideline-noncompliant manner. As another crucial point, the participants in our study recommended the expansion of medical home visits and treatments in specialised outpatient treatment centres to avoid complex and hazardous transport and stressful hospital stays. Moreover, continuity of care should be ensured by the establishment of a companionship by a trusted and professional adviser. International care models using medical home visits, case management and/or shared care approaches could be forward-looking with respect to developing new concepts to support tracheotomised and/or ventilated patients [50].

4. ***Involvement in decisions and respect for preferences***

Our study found that the living situation (i.e., PH vs. SLC) did not influence HRQL. This was in line with Huttmann et al. [13], who found no differences in HRQL between individuals with invasive ventilation living at home compared to those living in nursing facilities. This was convergent with our qualitative component revealing that it was possible to enable social participation with family and friends in both settings. The balance between dependence and independence (57 meaning units) was a central theme here. The importance of being accepted and living a normal live despite high dependency has previously been demonstrated in national and international studies [51–54]. Consequently, it is important to continuously reflect on one's own practice to ensure that one is actually actively listening and asking to learn about the ventilated person's preferences.

5. ***Clear information, communication, and support for self-care***

In the qualitative part of our study, being supported in developing knowledge and confidence was a central point for delivering person-centred care. In line with Dyrstad et al. [55], we found that the individuals who perceived themselves as well informed were more satisfied with treatment and decisions about their life situation. To support clear communication, we recommend sharing tailored information and using a trusted advisor to support decision-making.

6. ***Involvement of, and support for, family and carers***

Consistent with other studies [24, 30], we found a moderate to high burden for the majority of family caregivers (BSFC-s summary score: 12.81 ±8.74). We did not find any relevant difference in the BSFC-s summary score between family caregivers caring for someone at home and family caregivers caring for someone in an SLC (PH: 13.20 ±10.18 vs. SLC: 12.64 ±8.55). One would expect that caring for someone in their own home would be more of a burden than caring for a resident in an SLC. However, what seems surprising at first glance becomes conclusive on closer inspection of our qualitative findings (sub-code ‘dealing with burdens and challenges’: 69 meaning units). Ventilated individuals in the home setting usually receive 24/7 intensive care, which reduces the burden on family caregivers, particularly in terms of nursing care. Furthermore, ventilated individuals in an SLC are also supported by their family caregivers, e.g., by frequent and time-consuming visits, care coordination, managing finances, shopping or household activities. Other qualitative studies have confirmed a substantial burden to the lives of family caregivers and call for approaches to support them and improve caregiver well-being [24, 30, 31]. To reduce the burden on family caregivers and improve care overall, we recommend care focusing on the family, and not only the ventilated individual.

7. ***Emotional support, empathy and respect***

In agreement with other studies [26, 56], we found moderate scores for the emotional components of the SRI presented by the subscales ‘anxiety’ (64.40 ±24.87) and ‘psychological well-being’ (59.96 ±22.67). The qualitative component of our study showed that delivering person-centred care is possible but needs respect and understanding about the ventilated individuals and their families. Additionally, in line with other studies [23, 24, 31], we became aware of a challenging psychosocial factor: Due to the continuous presence of a nurse or an assistant, the boundaries between professional care and family become blurred. Therefore, we recommend to deepen psychosocial aspects in further education and to conduct regular supervisions.

8. ***Attention to physical and environmental needs***

Attentive physical care is one of the most important services that nurses can provide. According to our participants, ‘communication and being understood’ is considered a basic condition. Since people on HMV are often limited in their communication, there is a need for augmentative and alternative communication and attention to physical signals. Studies focusing on communication in individuals on HMV confirm this [57, 58].

Similar to our results, studies using the SRI for reporting HRQL in heterogeneous HMV patient groups have reported the most severe impairments in the domain ‘physical functioning’, which characterises the group of HMV users as a population with strong physical dependence, leading to complex care needs [13, 26, 56]. Additionally, well known from other studies [3, 7, 8], we found that the use of health-related resources is extensive and complex in individuals on HMV. In particular, the provision of ventilation equipment, medical aids and technical devices depends strongly on the individual needs of the ventilated person. Differences between the care settings could hardly be found. One exception was the powered wheelchair, which was used significantly more often in PHs than in SLCs (PH: 59.3 % vs. SLC: 5.9 %). This fact can be seen as a surrogate for participation since it enables the affected person to move independently. Convergent to this and in line with another study [52], the participants reported in our qualitative interviews that despite complex care needs, participation could be improved through technology. In this regard, the participants emphasised that there has to be a balance between safety and living an active life. Studies dealing with the safety of individuals on HMV concluded that the focus should not be solely on the clinical and technical aspects of safety but rather on the interpersonal aspects [23, 24]. Overall, it is important that the technologies used benefit the ventilated individuals and that they do not have to adapt to the technologies.

### Strengths and limitations

Our study had several important strengths. We conducted our convergent parallel mixed-methods study with a high degree of methodological rigour. The findings of the study benefit from the strengths of both methods used. As recommended for studies dealing with the multidimensional construct ‘quality of care’, we compared the perceived quality with the clinical quality [19]. Using the framework method [44] and the Picker principles [21, (Picker Institute Europe: Picker Principles of Person Centred Care, unpublished)] to guide our qualitative analysis and merge the qualitative and quantitative results, our findings present a holistic and person-centred view on the quality of care for individuals on HMV. In addition to a strong methodological basis, our results provide expertise from ventilated individuals with various underlying diseases, different types of ventilation and different living conditions. People with communicative impairments could take part in the interviews using communication devices, and people with cognitive impairment were able to participate in the study by proxy through a family caregiver. Furthermore, family caregivers were invited to present their own perception of quality of care in HMV.

Nevertheless, we have to discuss some limitations. Unfortunately, we were not able to recruit a high number of participants for our quantitative study component. In particular, recruitment via intensive care services was challenging. Of the 180 eligible patients, 22 ventilated individuals (response: 12.2 %) were available for study participation. Methodical challenges and low response rates are well known in research with vulnerable patient groups [59]. In addition, the COVID-19 pandemic made it even more challenging to conduct the study. However, due to the combination of various recruitment strategies, high flexibility, comprehensive resource input, and an additional online survey, we finally managed to recruit 46 participants for the quantitative component. Our qualitative sample (31 participants) was adequate to achieve meaningful saturation and to understand the bigger picture. Meaning saturation is usually reached after 16 to 24 interviews [46], but due to the high heterogeneity among our interview subjects, it seems understandable that our sample had to be somewhat larger. Moreover, based on the study by Karagiannidis et al. [3], it is likely that our sample consisted of participants who were less severely impaired than the average HMV population. Even though we tried to include individuals with cognitive and consciousness impairment in the study through their legal guardians and/or family caregivers, we were confronted with the phenomenon that people with a lack of capacity to informed consent are often excluded from study participation for protective reasons [60]. In addition, it must be taken into account that the perspectives of affected individuals and their family caregivers are different. However, studies show that proxy information tends to match well with self-reported information in surveys of health problems or care experiences [61, 62]. Furthermore, since access to participants was supported via intensive care services as gatekeepers, we have to consider the risk of selection bias. First, it can be assumed that intensive care providers who were convinced of the high quality of their services were more likely to support our study. Second, it cannot be ruled out that intensive care providers influenced participant selection by choosing patients who were more satisfied with their care situation. As recommended, we tried to avoid social desirability bias by assuring privacy and using techniques such as probing for more information [63]. Overall, this strategy seemed to succeed, as the participants appeared to be honest and transparent in their criticism.

## Conclusions

This convergent parallel mixed-methods study provides a holistic view of the multifaceted construct of quality of care for people on HMV. We could demonstrate that high-quality person-centred care is possible in both care settings. While people living at home tended to be younger and more independent, people living in SLCs were significantly older and more severely affected by their disease. HRQL and the burden of family caregivers were experienced as highly individually and independent of the living situation. The free choice of the care setting seems to have a strong influence on the perceived quality of care. Therefore, policies should facilitate person centred care, which includes the involvement of the ventilated individual in decisions about their care. In addition, we recommend a continuous self-reflection of one’s own person-centred attitude, the reduction of bureaucracy in order to relieve families, the support of professional nursing through the introduction of APNs, the promotion of home visits and outpatient checks-ups, clear communication to enable own decisions, a family centred care taking psychosocial aspects into account and the use of technologies to enable participation.

## Supplementary Information

**Additional file 1.** Codebook of the framework analysis.

**Additional file 2.** Further subgroup analysis.

## Data Availability

The datasets generated and analysed during the current study (interview transcripts and MAXQDA data) are not publicly available due to participant confidentiality considerations. Aggregate data are available from the corresponding author upon reasonable request.

## Abbreviations

ANP: Advanced Nursing Practice
BSFC-s: Burden Scale for Family Caregivers – short version
COPD: Chronic obstructive pulmonary disease
GRAMMS: Guidance for Good Reporting of a Mixed Methods Study
HRQL: Health-related quality of life
HMV: Home mechanical ventilation
ICU: Intensive care unit
ID: Identity number
IPReG: Intensive Care and Rehabilitation Strengthening Act
M: Mean
MDK: Medical Service of the Health Insurance Funds
NMD: Neuromuscular disorder
NIV: Non-invasive ventilation
OVER-BEAS: Optimising the Care of Ventilated Patients in Outpatient Intensive Care
PH: Private home
SD: Standard deviation
SHAPE: Safety in Home Care for Ventilated Patients study
SLC: Shared living community
SRI: Severe Respiratory Insufficiency questionnaire

## References

[1] Simonds AK (2016). Home Mechanical Ventilation: An Overview. Annals ATS..

[2] Lloyd-Owen SJ, Donaldson GC, Ambrosino N, Escarabill J, Farre R, Fauroux B (2005). Patterns of home mechanical ventilation use in Europe: results from the Eurovent survey. Eur Respir J..

[3] Karagiannidis C, Strassmann S, Callegari J, Kochanek M, Janssens U, Windisch W (2019). Epidemiologische Entwicklung der außerklinischen Beatmung: Eine rasant zunehmende Herausforderung für die ambulante und stationäre Patientenversorgung. [Evolving Epidemiology of Home Mechanical Ventilation: A Rapidly Growing Challenge for Patient Care]. Deutsche Medizinische Wochenschrift.

[4] Nasiłowski J, Wachulski M, Trznadel W, Andrzejewski W, Migdał M, Drozd W (2015). The evolution of home mechanical ventilation in poland between 2000 and 2010. Respir Care..

[5] Povitz M, Rose L, Shariff SZ, Leonard S, Welk B, Jenkyn KB (2018). Home Mechanical Ventilation: A 12-Year Population-Based Retrospective Cohort Study. Respir Care..

[6] Schwarz SB, Wollsching-Strobel M, Majorski DS, Magnet FS, Mathes T, Windisch W. The development of inpatient initiation and follow-up of home mechanical ventilation in Germany. DTSCH AERZTEBL INT. 2021. 10.3238/arztebl.m2021.0193. 10.3238/arztebl.m2021.0193 PMC8378259 34304756

[7] Lehmann Y, Ostermann J, Reinhold T, Ewers M (2019). Gesundheitsökonomische deskriptive Analyse der häuslichen Intensivversorgung beatmeter Patienten. [Descriptive Analysis of Health Economics of Intensive Home Care of Ventilated Patients]. Gesundheitswesen..

[8] Nonoyama ML, McKim DA, Road J, Guerriere D, Coyte PC, Wasilewski M, et al. Healthcare utilisation and costs of home mechanical ventilation. Thorax. 2018. 10.1136/thoraxjnl-2017-211138. 10.1136/thoraxjnl-2017-211138 29374088

[9] Windisch W, Geiseler J, Simon K, Walterspacher S, Dreher M (2018). German National Guideline for Treating Chronic Respiratory Failure with Invasive and Non-Invasive Ventilation - Revised Edition 2017: Part 2. Respiration..

[10] Windisch W, Geiseler J, Simon K, Walterspacher S, Dreher M (2018). German National Guideline for Treating Chronic Respiratory Failure with Invasive and Non-Invasive Ventilation: Revised Edition 2017 - Part 1. Respiration..

[11] Dellweg D (2011). Statuserhebung von Pflegediensten für außerklinische Beatmung. Survey of Nursing Services with Regard to Mechanical Ventilation at Home. Pneumologie..

[12] Windisch W, Dellweg D, Geiseler J, Westhoff M, Pfeifer M, Suchi S, Schönhofer B (2020). Prolonged Weaning from Mechanical Ventilation. DTSCH Aerztebl Int..

[13] Huttmann SE, Windisch W, Storre JH (2015). Invasive home mechanical ventilation: living conditions and health-related quality of life. Respiration..

[14] MacIntyre EJ, Asadi L, Mckim DA, Bagshaw SM (2016). Clinical Outcomes Associated with Home Mechanical Ventilation: A Systematic Review. Can Respir J..

[15] Horvath L, Böhm D, Gleich S (2019). Schwerpunktüberprüfung ambulanter Wohngemeinschaften der außerklinischen Intensivpflege im Stadtgebiet München – Ergebnisse und Rückschlüsse. [Surveillance of Supervised Flat-Sharing Communities Requiring Intensive Home Care: Results and Conclusions]. Gesundheitswesen.

[16] Köhler D (2019). Explosive Zunahme der häuslichen Krankenpflege bei Beatmeten und Tracheotomierten. [Tremendous Increase of Home Care in Ventilated and Tracheostomized Patients - Reasons, Consequences, Solutions]. Dtsch Med Wochenschr..

[17] Rosseau S (2017). Positionspapier zur aufwendigen ambulanten Versorgung tracheotomierter Patienten mit und ohne Beatmung nach Langzeit-Intensivtherapie (sogenannte ambulante Intensivpflege). [Tracheostomy Home Care of Patients after Long Term Ventilation on the ICU - a Position Paper]. Pneumologie..

[18] Reichardt A (2020). Ein Gesetzt mit vielen Schwächen. Deutsches Ärzteblatt..

[19] Hanefeld J, Powell-Jackson T, Balabanova D (2017). Understanding and measuring quality of care: dealing with complexity. Bull World Health Organ..

[20] Institute of Medicine (2009). Crossing the quality chasm: A new health system for the 21st century.

[21] Cleary PD, Edgman-Levitan S, Walker JD, Gerteis M, Delbanco TL (1993). Using patient reports to improve medical care: a preliminary report from 10 hospitals. Qual Manag Health Care..

[22] Larrabee JH, Bolden LV (2001). Defining patient-perceived quality of nursing care. J Nurs Care Qual..

[23] Schaepe C, Ewers M (2017). "I need complete trust in nurses" - home mechanical ventilated patients' perceptions of safety. Scand J Caring Sci..

[24] Schaepe C, Ewers M (2018). "I see myself as part of the team" - family caregivers' contribution to safety in advanced home care. BMC Nurs..

[25] Swedberg L, Chiriac EH, Törnkvist L, Hylander I (2013). From risky to safer home care: health care assistants striving to overcome a lack of training, supervision, and support. Int J Qual Stud Health Well-being..

[26] Huttmann SE, Magnet FS, Karagiannidis C, Storre JH, Windisch W (2018). Quality of life and life satisfaction are severely impaired in patients with long-term invasive ventilation following ICU treatment and unsuccessful weaning. Ann Intensive Care..

[27] Schwarz SB, Mathes T, Majorski DS, Wollsching-Strobel M, Kroppen D, Magnet FS, Windisch W (2021). Living conditions and autonomy levels in COPD patients receiving non-invasive ventilation: impact on health related quality of life. BMC Pulm Med..

[28] Cederwall C-J, Olausson S, Rose L, Naredi S, Ringdal M (2018). Person-centred care during prolonged weaning from mechanical ventilation, nurses' views: an interview study. Intensive Crit Care Nurs..

[29] Dybwik K, Tollåli T, Nielsen EW, Brinchmann BS (2011). "Fighting the system": families caring for ventilator-dependent children and adults with complex health care needs at home. BMC Health Serv Res..

[30] Evans R, Catapano M, Brooks D, Goldstein R, Avendano M (2012). Family caregiver perspectives on caring for ventilator-assisted individuals at home. Can Respir J..

[31] Limberger R, Schnepp W (2020). How Do Relatives Experience Advanced Home Care? A Qualitative Study. Home Health Care Manage Pract..

[32] Gerken L, Klingshirn H, Reuschenbach B (2020). Beatmete Menschen in der außerstationären Intensivpflege. Pflegezeitschrift..

[33] Klingshirn H, Gerken L, Hofmann K, Heuschmann PU, Haas K, Schutzmeier M (2021). How to improve the quality of care for people on home mechanical ventilation from the perspective of healthcare professionals: a qualitative study. BMC Health Serv Res..

[34] Creswell JW (2013). Qualitative inquiry and research design: Choosing among five approaches.

[35] Tariq S, Woodman J (2013). Using mixed methods in health research. JRSM Short Rep..

[36] Creswell JW (2015). A concise introduction to mixed methods research.

[37] O'Cathain A, Murphy E, Nicholl J (2008). The quality of mixed methods studies in health services research. J Health Serv Res Policy..

[38] Naumann E, Möhring K, Reifenscheid M, Wenz A, Rettig T, Lehrer R (2020). COVID-19 policies in Germany and their social, political, and psychological consequences. Eur Policy Anal..

[39] Reinhard H-J, Becker U, Reinhard H-J (2018). Long-Term Care in Germany. Long-Term Care in Europe: A Juridical Approach.

[40] Windisch W, Freidel K, Schucher B, Baumann H, Wiebel M, Matthys H, Petermann F (2003). The Severe Respiratory Insufficiency (SRI) Questionnaire A specific measure of health-related quality of life in patients receiving home mechanical ventilation. J Clin Epidemiol..

[41] Oga T, Windisch W, Handa T, Hirai T, Chin K (2018). Health-related quality of life measurement in patients with chronic respiratory failure. Respiratory investigation..

[42] Graessel E, Berth H, Lichte T, Grau H (2014). Subjective caregiver burden: validity of the 10-item short version of the Burden Scale for Family Caregivers BSFC-s. BMC Geriatr..

[43] Pendergrass A, Malnis C, Graf U, Engel S, Graessel E (2018). Screening for caregivers at risk: Extended validation of the short version of the Burden Scale for Family Caregivers (BSFC-s) with a valid classification system for caregivers caring for an older person at home. BMC Health Serv Res..

[44] Gale NK, Heath G, Cameron E, Rashid S, Redwood S (2013). Using the framework method for the analysis of qualitative data in multi-disciplinary health research. BMC Med Res Methodol..

[45] Elo S, Kyngäs H (2008). The qualitative content analysis process. J Adv Nurs..

[46] Hennink MM, Kaiser BN, Marconi VC (2016). Code Saturation Versus Meaning Saturation: How Many Interviews Are Enough?. Qual Health Res..

[47] Fetters MD, Curry LA, Creswell JW (2013). Achieving integration in mixed methods designs-principles and practices. Health Serv Res..

[48] Lehmann Y, Stark S, Ewers M (2020). Providing care to long-term mechanically ventilated patients in Germany – Current situation and needs for action from the perspective of health professionals. Int J Health Prof..

[49] Lehmann Y, Ewers M (2018). Wege invasiv beatmeter Patienten in die häusliche Beatmungspflege: Die Perspektive ambulanter Intensivpflegedienste. [Pathways of Invasive Ventilated Patients Released into Intensive Home Care: The Perspective of Home Care Providers]. Gesundheitswesen..

[50] Stark S, Ewers M (2020). Long-Term Care for Tracheotomised Patients With or Without Invasive Ventilation. Lessons Learned from a Scoping Review of International Concepts. Int J Integr Care..

[51] Dreyer PS, Steffensen BF, Pedersen BD. Living with severe physical impairment, Duchenne's muscular dystrophy and home mechanical ventilation. Int J Qual Stud Health Well-being. 2010. 10.3402/qhw.v5i3.5388. 10.3402/qhw.v5i3.5388 PMC2915819 20689774

[52] Israelsson-Skogsberg Å, Hedén L, Lindahl B, Laakso K (2018). 'I'm almost never sick': Everyday life experiences of children and young people with home mechanical ventilation. J Child Health Care..

[53] Nelissen V, Metzing S, Schnepp W (2019). What it means for people to be mechanically ventilated via a tracheostomy At home: a qualitative study. Cent Eur J Nurs Midwifery..

[54] Yamaguchi M, Suzuki M (2013). Independent living with Duchenne muscular dystrophy and home mechanical ventilation in areas of Japan with insufficient national welfare services. Int J Qual Stud Health Well-being..

[55] Dyrstad DN, Hansen BS, Gundersen EM (2013). Factors that influence user satisfaction: tracheotomised home mechanical ventilation users' experiences. J Clin Nurs..

[56] Budweiser S, Hitzl AP, Jörres RA, Schmidbauer K, Heinemann F, Pfeifer M (2007). Health-related quality of life and long-term prognosis in chronic hypercapnic respiratory failure: a prospective survival analysis. Respir Res..

[57] Laakso K, Markström A, Idvall M, Havstam C, Hartelius L (2011). Communication experience of individuals treated with home mechanical ventilation. Int J Language Commun Disord.

[58] Lindahl B, Sandman P-O, Rasmussen BH (2006). On Being Dependent on Home Mechanical Ventilation: Depictions of Patients' Experiences Over Time. Qual Health Res..

[59] Gnass I, Krutter S, Elsner F, Osterbrink J (2016). Methodische Herausforderungen bei der Rekrutierung vulnerabler Patienten. Palliativmedizin..

[60] Shepherd V, Wood F, Griffith R, Sheehan M, Hood K (2019). Protection by exclusion? The (lack of) inclusion of adults who lack capacity to consent to research in clinical trials in the UK. Trials..

[61] Dewey ME, Parker CJ (2000). for The Medical Research Council Cognitve Function and Ageing Study. Survey into health problems of elderly people: a comparison of self-report with proxy information. Int J Epidemiol..

[62] Zuckerbraun S, Allen RW, Flanigan T (2019). Paired Interviews to Evaluate Patient and Proxy Responses on Patient Experience of Care Surveys (PECS). Field Methods..

[63] Bergen N, Labonté R (2020). "Everything Is Perfect, and We Have No Problems": Detecting and Limiting Social Desirability Bias in Qualitative Research. Qual Health Res.

