# Supplementary material for: Comparing the quality of care for long-term ventilated individuals at home versus in shared living communities: a convergent parallel mixed-methods study
Source: BMC Nurs. 2022 Aug 11;21:224. doi: 10.1186/s12912-022-00986-z (PMC9368695; doi:10.1186/s12912-022-00986-z)
Supplement: Supplementary file 1 — Additional file 1. Codebook of the framework analysis. [file 12912_2022_986_MOESM1_ESM.pdf]

Additional file 1: Codebook of the framework analysis

This codebook provides an in-depth description of our analysis matrix. We used the framework method [1] to guide this qualitative content analysis [2]. The *Picker Principles of Person-Centred Care* [3, 4] were used as a theoretical structure for deductive content analysis (meta-codes). Sub-codes were built inductively.

The *Picker Principles of Person-Centred Care* have their origins in the Picker/Commonwealth Program in 1987. The Program aimed to promote measures of providing medical care that are sensitive to the needs and concerns of the patient [4].

The definitions of the *Picker principles* are cited verbatim from an unpublished report of the Picker institute [3]. Each sub-code is presented with a description of the content and two example quotes (i.e., private home vs. shared living community) of the interviews with the ventilated individuals and their family caregivers.

Contents

1. Fast access to reliable health advice 2
2. Effective treatment delivered by trusted professionals 3
3. Continuity of care and smooth transitions 4
4. Involvement in decisions and respect for preferences 5
5. Clear information, communication, and support for self-care 6
6. Involvement of, and support for, family and carers 7
7. Emotional support, empathy and respect 8
8. Attention to physical and environmental needs 9

References 10

<table>
<tr><td colspan="2">1. Fast access to reliable health advice</td></tr>
<tr><td colspan="2">Getting access to the right services at the right time is essential to receiving high quality care and treatment, and reduces the likelihood of people experiencing unmet needs. Access can be an issue at various points throughout people’s care journeys, including the ease or difficulty of scheduling appointments, the time spent waiting for referrals or admissions, and the availability of appropriate clinicians and advice. Speed of access matters in a crisis, too, where delays can lead to deteriorations in people’s conditions – and where a rapid response can be of vital importance.</td></tr>
<tr><td colspan="2">1.1 Being integrated in a safe health care network</td></tr>
<tr><td>Description</td><td>• Appropriate health care network.
• Direct contact to known and trusted advisers.
• Support from an interprofessional team.
• 24 hours emergency service.</td></tr>
<tr><td>Private home</td><td>Communication is usually very independent, [...] you try to plan what you can. So, if there are agreements, medical appointments, then, because [...] you know each other, [...] you simply call [...]. (Ventilated individual, ID08, §80)</td></tr>
<tr><td>Shared living community</td><td>If there's a problem, whether it's an item out of stock [...] or a technical problem [...], [the equipment provider] is here within two hours maximum. [...] Last year [...] [my father] wanted to go to a concert, [...] he immediately [...] provided an extra battery. (Family caregiver, ID20, §43)</td></tr>
<tr><td colspan="2">1.2 Coping with inappropriate health care structures</td></tr>
<tr><td>Description</td><td>• Desperate search for an appropriate care setting.
• Impeded access to therapeutic measures or interprofessional treatment.
• Accelerated transition.
• Few outpatient medical centres.</td></tr>
<tr><td>Private home</td><td>Well, I do all that myself. [...] In the past [we] always had physical therapy at home, [...] but it is always so difficult to plan. Occupational and speech therapy are not available anyway, and I usually do it myself, the movement or the respiratory therapy. (Family caregiver, ID22, §71)</td></tr>
<tr><td>Shared living community</td><td>There are institutions that have said they won't do it. And then there are [...] waiting lists [...]. And we were pressed for time, because [...] if we don't find anything, then [the clinic] will find us something random. He has to leave. [...] There was no treatment anymore. (Family caregiver, ID31, §19)</td></tr>
<tr><td colspan="2">1.3 Fighting for a high-quality care</td></tr>
<tr><td>Description</td><td>• Need for knowledge, experience, perseverance and strength.
• Fight for medical aids and assistive technologies is extremely burdensome.
• Flat-rate payments impair quality of care.</td></tr>
<tr><td>Private home</td><td>Certain areas [are] paid only as a lump sum by the health insurance. [...] The quality [...] [is] getting significantly worse [...] and [...] sometimes [you] really have to fight [...]. And not all family caregivers, not all patients can fight. (Family caregivers, PH, ID14, §91)</td></tr>
<tr><td>Shared living community</td><td>He doesn't have a wheelchair because the health insurance company has rejected it several times. [...] I have to apply for everything again, they are playing for time, that is my interpretation of things. (Family caregiver, ID15, §26)</td></tr>
</table>

**2. Effective treatment delivered by trusted professionals**	
Positive therapeutic relationships between patients and staff are at the heart of person centred care. People should receive the most appropriate and effective care for their needs and be treated in a way that recognises and respects the outcomes that matter most to them. Interactions with care professionals should inspire a sense of confidence and trust.	
2.1 Feeling comfortable with the personal care situation	
**Description**	• Trusted therapeutic relationship.<br>• Care is guided by the idea of assistance.<br>• Care is tailored to individual needs.
**Private home**	So, what is important to me about the nurses is - and most of them [...] also have this - patience, time, listening, doing what I say and doing things the way I need them and not the way they are used to or someone else dictates. (Ventilated individual, PH, ID03, §13)
**Shared living community**	The occupational therapist [is] extremely committed [...] – such reviewers come regularly [...] and when they come the [occupational therapist] also comes here [...] and takes care of these people, clarifies with them [...] what [my partner] needs and doesn't need. Or when I have to write an application to the health insurance company, she writes something down for me [...]. I find that very pleasant. (Family caregiver, ID15, §22)
2.2 Feeling between hope and reality – exploiting rehabilitation potentials	
**Description**	• Neglect of weaning and rehabilitation potentials.<br>• Discrepancy between desire and reality.<br>• Therapeutic goal setting.
**Private home**	I had imagined that I breathed spontaneously during the day via a ‘heat and moisture exchangers’ and only needed the support of a ventilator when lying down. In retrospect, it was a mistake that after the application of the tracheostomy tube, I spent 10 days as an inpatient for observation almost exclusively lying down with ventilation. During this period, the lungs stopped actively breathing - [...] irreversibly - and all efforts at ‘weaning’ were unsuccessful. This could have been done better. (Ventilated individual, ID02, §68)
**Shared living community**	At the beginning, I had the impression that he didn't really accept it. [...] He will always have to live with this tube, and that won't get better. Because I think the times of improvement are over. (Family caregiver, ID28, §39)
2.3 Feeling insecure due to staff and skills shortage	
**Description**	• Lack of qualified staff.<br>• High staff turnover.<br>• Many foreign educated nurses (language barriers and cultural differences).
**Private home**	For several years now, there have been many, many, many foreign nursing staff [...] who have difficulties with communication and understanding. Last night I wanted to change my legs somehow [...] and then they didn't understand at first, and I had to explain for five minutes what I wanted. And I try to say it in simple words. (Ventilated individual, ID01, §21)
**Shared living community**	[...] I just wish I would be able to go out more often alone with my husband in the wheelchair. But [...] [that's] not possible because I'm not really allowed to go alone [...]. And then there is just not enough staff [...] and so things like that don't happen. (Family caregiver, ID19, §157)

3. **Continuity of care and smooth transitions**	
Often, people's care journeys will bring them into contact with a range of care providers and health and social care staff. Providing a sense of continuity across these is vital to ensuring that people have good overall experiences. Continuity of care is not just influenced by the relationships people have with staff, but by how well information is shared between staff and services and by how organisations interact with one another.	
3.1 Moving into a world of uncertainty	
**Description**	• Discharge without support and consultation.<br>• Accelerated transition.
**Private home**	He spent three weeks in the hospital at the intensive care unit to be weaned form ventilation, and then we were discharged with the devices and lots of equipment and had to see how we could manage it. We were well provided with medical aids. [...] But it is simply a total change, also for the whole family. [...] (Family caregiver, ID22, §41)
**Shared living community**	We had to leave [the clinic] at that time, and that was [...] the first slap in the face. They said: in 14 days we need a place to care for our father. [...] But they said they had already called everywhere [...] but there was nothing available for him. [...] And we were pressed for time, because [the clinic] told us that if we couldn't find anything, they would find us something. He has to leave. (Family caregiver, SLC, ID31, §14-19)
3.2 Welcoming visitors in the safety of the own home	
**Description**	• Avoid complex and hazardous transports and stressful hospital stays.<br>• Need for medical home visits.<br>• Need for specialised outpatient treatment centres.
**Private home**	Since a medical centre for people with disabilities has been opened in my hospital and an inpatient stay is always quite time-consuming for me, we now do the ventilation control on an outpatient basis. I have a device at home for determining the nocturnal CO_2 readings, so I can bring information about the nocturnal course to the outpatient appointment. (Ventilated individual, ID02, §62)
**Shared living community**	It's about transport. Last time we had to wait almost six or even more hours for transport. [...] At the hospital, [...] to be transported back again. And [the intensive care service] said they can't provide nursing staff for the whole day - they have a lot of work here. [...] (Family caregiver, ID29, §50-56)
3.3 Going on a care journey in good company	
**Description**	• Providing a sense of continuity.<br>• Companionship by trusted professionals.<br>• Assistance with the change of sectors (e.g., hospital stays).
**Private home**	A circumstance that is really scandalous is [...], that if I have to go to the hospital, [...] the intensive care service is not allowed to bill me for any services because I am so well cared for in hospital. And that is 1000 % out of touch with reality. In the hospital there is no staff at all, [...] secondly, they have no idea how to deal with disabilities [...]. So, when I am in hospital, I simply need a nurse who knows me [...] at least during the day. (Ventilated individual, ID03, §63)
**Shared living community**	At the moment [...]the visiting general practitioners change frequently. I would like to see a bit more continuity. (Family caregiver, ID15, §117-119)

4. Involvement in decisions and respect for preferences	
People have the right to be involved in their health and care. Involvement in care supports people to play an active role and to feel more confident in considering treatment options, associated benefits and risks, and in making informed decisions. Care should be delivered in a way that is sensitive to the needs and preferences of the person. Focusing on the patient as an individual includes treating the person with respect and with sensitivity to their background, social and cultural values. Active listening and enquiry can be important to elicit people's preferences, which should not be assumed.	
4.1 Balancing dependence and independence	
**Description**	• Living a normal life despite dependence on machines and people.<br>• Making own decisions balancing benefits and risks.<br>• Being accepted and getting to equal or feeling incapacitated and excluded.
**Private home**	The intensive care service is always there: 24 hours. And my partner is always there, too. [...] [For example, we were once] at my cousin's. That was a day trip. [...] Or shopping in [the city]. (Ventilated individual, ID11, §25-27)
**Shared living community**	We go on excursions. For example, we once went on holiday to Lake Chiemsee for ten days. [...] There were three of a us. And there was a nurse who also drove us. (Ventilated individual, ID04, §55-57)
4.2 Feeling accepted with own needs and preferences	
**Description**	• Respect and interest for people's preferences.<br>• Support in recreation and leisure activities.<br>• Care life balance.
**Private home**	Fortunately, I have an optimal social environment - family, friends and acquaintances. I imagine that it is a challenge to communicate with me and also to be able to put up with my "external appearance", so I am happy that those around me can cope with it. Thanks to my wife, family and carers, I am also able to go on excursions and even holidays. (Ventilated individual, ID02, §43)
**Shared living community**	We meet once a week, in the afternoon, and talk about what we're going to eat the next week [...]. And then we can write our shopping list. Then we go shopping. I always come along [...]. And we are allowed to decide for ourselves what the service staff cook for us [...]. (Ventilated individual, SLC, ID06, §145)
4.3 Enabling time for relationships with family and friends	
**Description**	• Enabling activities and participation with family and friends.<br>• Visit and Welcome family and friends.
**Private home**	If I need support to visit friends, we arrange that my friends come and pick me up right here in the flat. Or I try to organise private assistants who then accompany me. (Ventilated individual, ID03, §31)
**Shared living community**	My mum, my dad, my brother and my husband go with me. And they always visit me there [...] Every now and then my friend [...] comes to visit. Yes, that's it. [...] (Ventilated individual, ID07, §85-87)

<table>
<tr><th colspan="2">5. Clear information, communication and support for self-care</th></tr>
<tr><td colspan="2">People using health and care services should receive reliable, high quality and accessible information at every stage of their journey. How and when information is communicated can be as important as the message. Any assessment of information needs should consider not only content, but also how and when information is provided. By ensuring that the way information is communicated is tailored to the individual, it means that people are better placed to understand and make informed decisions about their care. Support should be provided by staff that allows people to develop the knowledge, confidence and skills required for self-care.</td></tr>
<tr><td colspan="2">5.1 Being supported in developing knowledge and confidence</td></tr>
<tr><td>Description</td><td>• Access to individual, tailored information at every stage of the care journey.
• Enabling informed decision making.
• Having a trusted advisor.</td></tr>
<tr><td>Private home</td><td>For my wife, it is really existential that everything is right, in all respects. That's why she and I attach great importance to our nursing team being open, yes, and talking and being able to talk to us about everything, because that simply has to be, because obviously they also break through the highly personal area. And [...] you simply need the necessary trust. (Family caregiver, ID23, §31)</td></tr>
<tr><td>Shared living community</td><td>When [my father] was to be discharged, the social services at the hospital helped me a lot with the formalities and told me that there were SLC specialised for intensive care, which I didn't know before. (Family caregiver, SLC, ID24, §87)</td></tr>
<tr><td colspan="2">5.2 Being alone with questions and decisions</td></tr>
<tr><td>Description</td><td>• No empowerment to make informed decisions.
• No support in developing knowledge, confidence and skills for self-care.</td></tr>
<tr><td>Private home</td><td>There is a lack of support from the health insurance [...] For example, I reported the [staffing] bottlenecks that we are experiencing at the moment to the health insurance at an early stage. What I got were two internet addresses where you could find care services. And then I was supposed to become active. (Family caregiver, ID14, §95)</td></tr>
<tr><td>Shared living community</td><td>These 13 devices that constantly run around the clock cost [...] electricity. And health insurance pays for it. I didn't know that. No one told me either. [...] Thank God, our state is there, but you have to know how the state can help us. But how should I know that? (Family caregiver, ID27, §117)</td></tr>
</table>

6. Involvement of, and support for, family and carers	
Providers and staff should acknowledge the importance of people's families, carers, and friends in their overall health and wellbeing, and should welcome their involvement. This includes providing support to help family members and carers to assist patients during and after care. Equally, the emotional impact that caring responsibilities have on family members and carers should not be underestimated; the ability to access support and to speak to staff about worries is important.	
6.1 Being ripped out of life and returning to normal	
**Description**	• Being ripped out of life, reorientation, and returning to a normal life.<br>• Support family members in being mindful and speaking about worries.
**Private home**	I'm glad that I can handle everything and that we have meanwhile adjusted our everyday life to it so that [...] [the whole family] can cope with it quite well. [...] It is a stroke of fate in the end, right, for everyone involved. (Family caregiver, ID22, §36)
**Shared living community**	It was a completely different life. When I came home, there was a hot meal on the table. And from then on, I had free time, so to speak. [...] We were together a lot at home, I had a small garden and a terrace with lots of flowers, I gave all that up, too. [...] And also [...] my personality. So, these values that were valid for me before, they just don't apply anymore. And I'm on the search again. (Family caregiver, ID15, §48-50)
6.2 Caring hand in hand	
**Description**	• Clear roles and responsibilities.<br>• Handing over control versus keeping control.
**Private home**	So, for the most part I take care of my medical aids [...] by myself. I make sure that I have what I need. [...] And the nursing part, doctors, examinations etc., that is actually organised [by the care service] when it is due. (Ventilated individual, ID08, §76)
**Shared living community**	[The coordination of care] is collaborative: [...] We as relatives [...] with the nurse. [...] If certain physicians [...] drop out, then we start searching again. [...] The [nursing staff] tend to make the appointments, because they are in everyday life, they know how it fits best for them. And then it's always a symbiosis. (Family caregiver, ID31, §61-63)
6.3 Being part of a family – being cared for	
**Description**	• Being responsible for a family member as a matter of course.<br>• Welcome the involvement of family members and friends.
**Private home**	Some days are difficult. Some days, not always. But I am pleased that [my partner] is at home, that is the very best thing for me. [...] Love is love. And you don't throw a person away. (Family caregiver, ID26, §23-25)
**Shared living community**	When I was little, when I needed help myself, [my parents] were always there for me. And now, as I said, that my father is no longer able to take care of himself, he simply needs the appropriate help from his children [...]. And it doesn't matter to me personally at what time of day or night [...], if he needs help, then I give him this help, without ifs and buts. (Family caregiver, ID20, §25)

6.4 Dealing with burdens and challenges	
**Description**	• Care determines daily life (e.g., high workload, high bureaucracy, lack of flexibility, change in social life, financial burden, restrictive housing conditions).<br>• Feeling vulnerable and powerless (e.g., emotional impact, fears and worries, physical strains).
**Private home**	At the end of the day, it's always a balance between relief through the care service and privacy. And sometimes it's difficult to know what you need more. So, spontaneity, going out with friends, always presupposes that I also have someone for my son. And that's not in my life now, it has to be planned well in advance or you just have to be lucky that someone is available at short notice, which tends to be the exception. (Family caregiver, ID18, §13)
**Shared living community**	So, leisure time has become very, very little. What I sometimes allow myself is to disappear here for half an hour or three quarters of an hour and go into town and eat a piece of cake or [...] go for a walk. [...] I'm here [in the SLC] for four hours every day. (Family caregiver, SLC, ID15, §28)

**7. Emotional support, empathy and respect**	
To deliver person centred care, a caring holistic approach that includes the provision of support and empathy is needed. For care to be compassionate, it must be delivered with respect, dignity, sensitivity and with an understanding about the person.	
7.1 Blurring boundaries	
**Description**	• Maintain a professional distance.<br>• Being a professional and being part of a family.
**Private home**	So, I have [...] [everyday] assistance, that's an assistant, companion, so to speak, but also friendship [...] it depends on how you get along with each other. (Ventilated individual, ID08, §12)
**Shared living community**	The nurses do everything they have to do. [...] I am also very pampered by some of them. Not all of them, but some. Because I also have a nurse for whom I have a [loving] nickname. (Ventilated individual, ID06, §73-83)
7.2 Living with a shadow	
**Description**	• Between monitoring for safety and having no privacy.<br>• Person-centred attitude – living the idea of assistance.<br>• Care is delivered with respect and understanding about the person.
**Private home**	[My social relationships are] actually quite good, but also [limited], so I would say 85 % good [...]. But because I always have a shadow behind me, a nurse or someone else, it is of course hindered. (Ventilated individual, PH, ID01, §41)
**Shared living community**	The team [...] is [...] friendly. The way they deal with the patients [...] [is] for me [...] a winner of the lottery. [...] Everyone, [says] hello, they come in and say what they're doing [...] and laugh and make a joke sometimes, and that's just nice. (Family caregiver, ID31, §33-35)

7.3 Feeling isolated and defenceless	
**Description**	• Being dependent on others.<br>• Loss of dignity and respect - abuse of power.
**Private home**	Likewise, no consideration is given to whether you have to go to work yourself. [...] If you can' t work, [...] then you are very quickly somewhere at the bottom. [...] If you don't have support for your relative, you can't go to work, because who lets a relative die, nobody does. (Family caregiver, ID14, §95)
**Shared living community**	[My relative] is a loner and he doesn't want much social contact. And [...] [once] he might not have reacted or [...] [didn't do it] the way the nurses want him to do it, and then they turned off the TV for him. [...] As a punishment [...]. He only has the TV and nothing else. And I have to say that's not acceptable. (Family caregiver, ID25, §53-55)

**8. Attention to physical and environmental needs**	
Physical care that comforts people is one of the most essential services that staff can provide. This ranges from pain management to assistance with activities and daily living needs. The surroundings and environment in which people are being cared for should also be considered, including the ability to provide care in privacy, in a clean and comfortable setting, and which allows appropriate accessibility for visits by family and carers.	
8.1 Communicating and being understood	
**Description**	• Nonverbal or technology assisted communication.<br>• Listen, be patient and pay attention to physical signals.
**Private home**	After waking up, I have my communication device put into operation so that communication with the nursing staff and others is possible, and I can check e-mails, what's app, etc. (Ventilated individual, ID02, §26)
**Shared living community**	Non-verbal, so if I ask, for example, are you in pain, then I get a wink or a slight shake of the head or something. The answers that I need from [my father] in the respective situation definitely come. [...] Then there's sometimes a smile, or when he wants to tease me, he sticks his tongue out at me or something. The communication is on a slightly different level. (Family caregiver, ID20, §31)
8.2 Dealing with complex care needs and complex planning	
**Description**	• Complex physical care needs.<br>• Living in a highly technical environment.<br>• Every activity requires complex planning.
**Private home**	It's always a bit more difficult to organise. [...] I [cannot] drive alone with [my son] [...] because I cannot drive and do suction at the same time. [...] We [are] then very limited in winter because [...] the equipment manufacturers do not give a guarantee for temperatures below 5 degrees Celsius. [...] That's why meetings with friends usually take place at our place, because a lot of things are easier to manage there. We have accessibility here, we have no problems with going to the toilet. [...] Also with public transport, [it is] sometimes very complicated, especially when it comes to changing lines. (Family caregiver, ID14, §19)
**Shared living community**	[I'm] going [...] to the cinema or out for dinner sometimes. But I can't go somewhere and stay away for a few days [...]. That's not possible, of course. But I can stay away for hours at a time. (Ventilated individual, ID06, §115)

8.3 Improving participation through technology	
**Description**	• Accessibility and non-accessibility.<br>• Provision with medical aids and assistive devices.<br>• Being active through technology or inactive through missing technology.
**Private home**	[The battery of the ventilator] usually lasts four hours. Until now, that was always too little. [...] [I then] had to go home [...] because the ventilator no longer kept up. And then you can [...] get additional batteries, [...] then the time is increased to eight to ten hours, and certain things are possible. You can be out and about all day. (Ventilated individual, ID08, §90)
**Shared living community**	Soon I'll get a power wheelchair and they'll put a device on it where I can take the PEG and oxygen with me. [...] Then I can paint the town red. (Ventilated individual, ID05, §136-138)
8.4 Balancing safety and living an active life	
**Description**	• Safety management and prevention of critical incidents.<br>• No risk, no fun.
**Private home**	[There are], of course, a variety of 'risks' - starting [...] with a sudden failure of the ventilation on the way, up to some barriers- actually small, but nevertheless insurmountable with the wheelchair - that ultimately force you to turn around. But: 'No risk- no fun'. (Ventilated individual, PH, ID02, §49-50)
**Shared living community**	For example, when you go outside, and it's lovely and warm here, if a nurse comes along, I feel safe. (Ventilated individual, ID07, §69)

References

1. Gale NK, Heath G, Cameron E, Rashid S, Redwood S. Using the framework method for the analysis of qualitative data in multi-disciplinary health research. BMC Med Res Methodol. 2013;13:117. doi:10.1186/1471-2288-13-117.
2. Elo S, Kyngäs H. The qualitative content analysis process. J Adv Nurs. 2008;62:107–15. doi:10.1111/j.1365-2648.2007.04569.x.
3. Picker Institute Europe. Picker Principles of Person Centred Care. Internal Picker Institute Europe report: unpublished; 2018.
4. Cleary PD, Edgman-Levitan S, Walker JD, Gerteis M, Delbanco TL. Using patient reports to improve medical care: a preliminary report from 10 hospitals. Qual Manag Health Care. 1993;2:31–8.
