# Supplementary material for: Comparing the quality of care for long-term ventilated individuals at home versus in shared living communities: a convergent parallel mixed-methods study
Source: BMC Nurs. 2022 Aug 11;21:224. doi: 10.1186/s12912-022-00986-z (PMC9368695; doi:10.1186/s12912-022-00986-z)
Supplement: Supplementary file 2 — Additional file 2. Further subgroup analysis. [file 12912_2022_986_MOESM2_ESM.pdf]

## **Additional file 2:** Further subgroup analysis

**Table A1** Demographic and ventilation characteristics in the invasive versus non-invasive groups

| | **Invasive** | | **NIV** | | **P value** | **P adjusted [a]** |
|---|---|---|---|---|---|---|
| Subjects, N | 37 | | 9 | | | |
| Gender, n (%) | | | | | .139 | .293 |
| Female | 13 | (35.1) | 4 | (44.4) | | |
| Male | 24 | (64.9) | 4 | (44.4) | | |
| Divers | 0 | 0 | 1 | (11.1) | | |
| Age, years, M (SD) | 55.02 | (±16.96) | 49.76 | (±15.15) | .400 | .401 |
| Underlying disease, n (%) | | | | | .098 | .293 |
| NMD | 16 | (43.2) | 6 | (66.7) | | |
| Spinal cord paralysis | 4 | (10.8) | 0 | 0 | | |
| CNSD | 8 | (21.6) | 0 | 0 | | |
| COPD | 5 | (13.5) | 1 | (11.1) | | |
| Pneumonia | 2 | (5.4) | 0 | 0 | | |
| Chest deformity | 0 | 0 | 2 | (22.2) | | |
| Post-OP complications | 2 | (5.4) | 0 | 0 | | |
| Living situation, n (%) | | | | | .069 | .277 |
| PH | 20 | (54.1) | 8 | (88.9) | | |
| SLC | 17 | (45.9) | 1 | (11.1) | | |
| Duration of ventilation, years, | 7.90 | (±8.10) | 12.06 | (±5.15) | .050 | .249 |
| M (SD) [b] | 5.22 | (0.14-32.96) | 13.45 | (1.99-17.26) | | |
| Spontaneous breathing, n (%) | | | | | <.001* | .002* |
| Ventilation < 16 h | 9 | (24.3) | 8 | (88.9) | | |
| Ventilation > 16 h | 5 | (13.5) | 1 | (11.1) | | |
| Continuous ventilation | 23 | (62.2) | 0 | 0 | | |

***N = 46***. Data are presented as absolute numbers (n) and relative frequencies (%) or as means (M) and standard deviations (SD).

*****Significant at $p < 0.05$.

[a] Adjusted with Bonferroni–Holm correction for multiple testing.

[b] Non-normally distributed data is also presented with media, minimum, and maximum values.

***Missing values:*** duration of ventilation, years (n = 3)

***Abbreviations:*** chronic obstructive pulmonary disease (COPD), central nervous system disease (CNSD), neuromuscular disorder (NMD), non-invasive ventilation (NIV), operative (OP), private home (PH), shared living community (SLC).

**Table A2** Health-related quality of life in the invasive versus non-invasive groups

| | Invasive | | NIV | | P value | P adjusted [a] |
|---|---|---|---|---|---|---|
| | M | (±SD) | M | (±SD) | | |
| Respiratory Complaints | 65.68 | (±19.58) | 75.00 | (±18.31) | .276 | .827 |
| Physical Functioning | 21.65 | (±18.81) | 39.29 | (±24.75) | .051 | .307 |
| Attendant Symptoms and Sleep | 65.18 | (±18.70) | 67.86 | (±22.59) | .752 | >.999 |
| Social Relationships | 58.91 | (±21.78) | 76.79 | (±13.79) | .051 | .307 |
| Anxiety | 63.75 | (±27.77) | 66.43 | (±13.45) | .735 | >.999 |
| Psychological Well-Being | 56.03 | (±22.67) | 73.41 | (±18.06) | .074 | .307 |
| Social Functioning | 42.18 | (±22.16) | 64.29 | (±21.11) | .027 | .216 |
| SRI Summary Score | 52.65 | (±13.92) | 66.15 | (±13.83) | .036 | .254 |

***N = 31***. All data are presented as means (M) and standard deviation (SD).

*Significant at $p < 0.05$.

[a] Adjusted with Bonferroni–Holm correction for multiple testing.

***Missing values:*** respiratory complaints (n = 2), social relationships (n = 1), anxiety (n = 2), social functioning (n = 1), SRI summary score (n = 4).

***Abbreviations:*** non-invasive ventilation (NIV), Severe Respiratory Insufficiency questionnaire (SRI).

**Table A3** Demographic and ventilation characteristics in the offline versus online groups

| | Offline | | Online | | P value | P adjusted [a] |
|---|---|---|---|---|---|---|
| Subjects, N | 25 | | 21 | | | |
| Gender, n (%) | | | | | .439 | .877 |
| Female | 8 | (17.4) | 9 | (19.6) | | |
| Male | 17 | (37.0) | 11 | (23.9) | | |
| Divers | 0 | 0 | 1 | (2.2) | | |
| Age, years, M (SD) | 57.10 | (±17.80) | 50.29 | (±14.61) | .168 | .673 |
| Underlying disease, n (%) | | | | | .006 | .039* |
| NMD | 10 | (21.7) | 12 | (26.1) | | |
| Spinal cord paralysis | 0 | 0 | 4 | (8.7) | | |
| Central nervous system disease | 8 | (17.4) | 0 | 0 | | |
| COPD | 3 | (6.5) | 3 | (6.5) | | |
| Pneumonia | 2 | (4.3) | 0 | 0 | | |
| Chest deformity | 1 | (2.2) | 1 | (2.2) | | |
| Post-operative complications | 1 | (2.2) | 1 | (2.2) | | |
| Living situation, n (%) | | | | | <.001* | <.001* |
| PH | 8 | (17.4) | 20 | (43.5) | | |
| SLC | 17 | (37.0) | 1 | (2.2) | | |
| Type of ventilation, n (%) | | | | | | |
| Invasive | 22 | (47.8) | 15 | (32.6) | .264 | .792 |
| NIV | 3 | (6.5) | 6 | (13.0) | | |
| Duration of ventilation, years, M (SD) | 6.86 | (±6.31) | 10.57 | (±8.80) | .119 | .594 |
| Spontaneous breathing, n (%) | | | | | .920 | .920 |
| Ventilation < 16 h | 10 | (21.7) | 7 | (15.2) | | |
| Ventilation > 16 h | 3 | (6.5) | 3 | (6.5) | | |
| Continuous ventilation | 12 | (26.1) | 11 | (23.9) | | |

***N = 46***. Data are presented as absolute numbers (n) and relative frequencies (%) or as means (M) and standard deviations (SD).

*****Significant at $p < 0.05$.

[a] Adjusted with Bonferroni–Holm correction for multiple testing.

***Missing values:*** duration of ventilation, years (n=3)

***Abbreviations:*** chronic obstructive pulmonary disease (COPD), neuromuscular disorder (NMD), non-invasive ventilation (NIV), private home (PH), shared living community (SLC).

**Table A4** Health-related quality of life in the offline versus online groups

| | Offline | | Online | | P value | P adjusted [a] |
|---|---|---|---|---|---|---|
| | **M** | **(±SD)** | **M** | **(±SD)** | | |
| Respiratory Complaints | 74.83 | (±15.78) | 58.16 | (±20.41) | .020 | .157 |
| Physical Functioning | 29.48 | (±19.56) | 22.92 | (±23.75) | .385 | >.999 |
| | 29.17 | (0-66.67) | 20.83 | (0-75.00) | | |
| Attendant Symptoms and Sleep | 68.06 | (±18.02) | 62.64 | (±21.21) | .449 | >.999 |
| Social Relationships | 63.04 | (±25.18) | 63.14 | (±16.21) | .990 | >.999 |
| Anxiety [b] | 70.94 | (±25.18) | 56.35 | (±22.88) | .107 | .748 |
| | 80 | (30-100) | 60 | (5-90) | | |
| Psychological Well-Being | 60.67 | (±23.49) | 58.97 | (±22.40) | .841 | >.999 |
| Social Functioning | 47.69 | (±23.39) | 46.88 | (±24.74) | .927 | >.999 |
| SRI Summary Score | 59.84 | (±12.81) | 51.55 | (±16.56) | .155 | .928 |

***N = 31***. All data are presented as means (M) and standard deviation (SD).

*****Significant at $p < 0.05$.

[a] Adjusted with Bonferroni–Holm correction for multiple testing.

[b] Non-normally distributed data is also presented with median, minimum, and maximum values.

***Missing values:*** respiratory complaints (n = 2), social relationships (n = 1), anxiety (n = 2), social functioning (n = 1), SRI summary score (n = 4).

***Abbreviations:*** Severe Respiratory Insufficiency questionnaire (SRI).
